# Myogenesis defects in a patient-derived iPSC model of hereditary GNE myopathy

**DOI:** 10.1038/s41536-022-00238-3

**Published:** 2022-09-09

**Authors:** Rebecca E. Schmitt, Douglas Y. Smith, Dong Seong Cho, Lindsey A. Kirkeby, Zachary T. Resch, Teerin Liewluck, Zhiyv Niu, Margherita Milone, Jason D. Doles

**Affiliations:** 1 Department of Biochemistry and Molecular Biology, Mayo Clinic, Rochester, MN 55905 USA; 2 Department of Anatomy, Cell Biology, and Physiology, Indiana University School of Medicine, Indianapolis, IN 46202 USA; 3 Indiana Center for Musculoskeletal Health, Indianapolis, IN 46202 USA; 4 Center for Regenerative Medicine, Mayo Clinic, Rochester, MN 55905 USA; 5 Department of Neurology, Mayo Clinic, Rochester, MN 55905 USA; 6 Department of Laboratory Medicine, Mayo Clinic, Rochester, MN 55905 USA

**Keywords:** Mechanisms of disease, Metabolic disorders

## Abstract

Hereditary muscle diseases are disabling disorders lacking effective treatments. UDP-N-acetylglucosamine-2-epimerase/N-acetylmannosamine kinase (GNE) myopathy (GNEM) is an autosomal recessive distal myopathy with rimmed vacuoles typically manifesting in late adolescence/early adulthood. *GNE* encodes the rate-limiting enzyme in sialic acid biosynthesis, which is necessary for the proper function of numerous biological processes. Outside of the causative gene, very little is known about the mechanisms contributing to the development of GNE myopathy. In the present study, we aimed to address this knowledge gap by querying the underlying mechanisms of GNE myopathy using a patient-derived induced pluripotent stem-cell (iPSC) model. Control and patient-specific iPSCs were differentiated down a skeletal muscle lineage, whereby patient-derived GNEM iPSC clones were able to recapitulate key characteristics of the human pathology and further demonstrated defects in myogenic progression. Single-cell RNA sequencing time course studies revealed clear differences between control and GNEM iPSC-derived muscle precursor cells (iMPCs), while pathway studies implicated altered stress and autophagy signaling in GNEM iMPCs. Treatment of GNEM patient-derived iMPCs with an autophagy activator improved myogenic differentiation. In summary, we report an in vitro, iPSC-based model of GNE myopathy and implicate defective myogenesis as a contributing mechanism to the etiology of GNE myopathy.

## Introduction

UDP-N-acetylglucosamine-2-epimerase/N-acetylmannosamine kinase (GNE) myopathy is an early adulthood onset, autosomal recessive, rare myopathy with a prevalence of ~1–9/1,000,000^1^. Some communities, such as the Persian Jewish community, have a much higher prevalence of ~1/1500^2^. Historically, prior to the identification of the underlying genetic defect, GNE myopathy was independently described by various investigators as Nonaka distal myopathy, distal myopathy with rimmed vacuoles, vacuolar myopathy sparing quadriceps, and inclusion body myopathy 2 (IBM2)^3–6^. On a molecular level, the *GNE* gene encodes a protein containing two enzymatic domains, the epimerase, and kinase domain, which are involved in sequential steps of the biosynthesis of sialic acid. Sialic acids are a common terminal sugar on glycolipids and glycoproteins and are involved in diverse biological functions from aging to immune responses^7^. To date, more than 200 mutations have been found in patients suffering from GNE myopathy (GNEM), and these mutations can be either homozygous, as seen in specific ethnic populations, or compound heterozygous, and occurring in either the same or different domains of GNE^1^. Clinically, GNEM manifests with distal lower limb weakness resulting in foot drop. Over time, the muscle weakness spreads to proximal muscles and eventually to the upper limb muscles but classically spares the quadriceps. Within two decades from disease onset, patients frequently have impaired mobility leading to a need for wheelchair assistance^4,8^. Pathologically, GNEM is characterized by rimmed vacuoles, congophilic inclusions, protein aggregates, and enhanced lysosomal activity as evidenced by increased acid phosphatase reactivity^1,8,9^.

There are currently no effective treatments for GNEM. Numerous organismal GNEM models have been developed to address this issue; however, most of these models have significant limitations that restrict their ability to provide insights into disease mechanisms or treatments. Several are briefly discussed below. A significant concern is that two of the three murine GNEM models simply do not develop or allow for the study of GNEM muscle hallmarks (e.g., rimmed vacuoles) or myopathic features typical of the human disease (e.g., muscle weakness)^10,11^. For example, some GNEM models feature glomerulopathy, which is not found in GNEM patients^10–12^. Concerns aside, there is one mouse model combining a *GNE* null allele with transgenic expression of a human GNE D176V allele that appears to rise above the rest. This mouse develops clinical characteristics comparable to those observed in GNEM patients^13^. For example, rimmed vacuoles, increased acid phosphatase reactivity, and increased Lysosomal Associated Membrane Protein 1 (LAMP1) and 2 (LAMP2) staining are evident in cross-sectional analyses of the gastrocnemius muscle. Furthermore, these mice display a loss of motor strength after the age of 30 weeks. As GNE null mice are embryonic lethal^10,14^, maintaining this model and generating experimental mice is challenging as the required mating scheme results in only 9% of the pups having the genotype of interest and have decreased survival over time^13^. Thus, while potentially useful for studying GNE disease etiology, this model is likely ill-suited for drug screening and/or medium- to large-scale preclinical intervention studies. In addition to murine models, GNEM has been also modeled using zebrafish^15^. Human and zebrafish GNE share the same functional domains and have a 90% similarity. In one study, morpholino-mediated GNE knockdown resulted in variable morphological deformities, ranging from slight to severe regarding overall size and tail/trunk development. Additionally, their skeletal muscle myofibers were found to be highly disorganized, a finding that likely explains the decreased larval locomotor activity in the normal/less severely affected GNE knockdown larvae compared to controls^15^. Unfortunately, despite the promise of these preliminary observations, little has since been pursued using this zebrafish model. This is particularly true with respect to mechanisms downstream of mutated GNE that could contribute to these muscle phenotypes or in the context of drug screening to mitigate the phenotype, which is one strength of the zebrafish system.

The role of mutated GNE in the development of muscle weakness and human myopathology is largely unknown. Several past and current clinical human trials using sialic acid, or its precursors, to treat GNEM have yielded underwhelming results or lack proper controls (i.e., baseline characteristics of control subjects were not matched with participants)^16,17^. These results raise the likely probability that GNE has additional functions beyond its role in sialic acid metabolism that are relevant to disease development and progression. Revealing novel mechanistic insights will require a model that reliably recapitulates the human phenotype, is feasible to obtain and is cost-effective. In this study, we report the development of an in vitro *GNE* myopathy model using patient-derived induced pluripotent stem cells (iPSCs). Utilizing this model, we identify previously unrecognized deficiencies in myogenesis. We corroborate these observations using longitudinal single-cell RNA sequencing analyses. Finally, we identified GNEM-associated alterations in stress and autophagy signaling that, when pharmacologically targeted, improved myogenesis. These data underscore the potential of iPSC-based models as a powerful discovery platform to deepen mechanistic understanding of disease as well as to predict and test novel therapies aiming to improve GNEM pathophysiology.

## Results

### GNE myopathy (GNEM) patient clinical phenotype and pathological evaluation of muscle biopsies

**Fig. 1:**
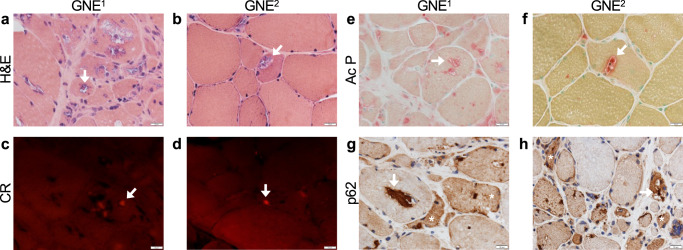
Characterization of GNEM patient muscle biopsies. **a**, **b** Hematoxylin and eosin (H&E) stained tissue cross-sections of two patients with GNE myopathy, GNE^1^ (left) and GNE^2^ (right), exhibit rimmed vacuoles (arrows) and muscle fiber size variability. **c**, **d** Congo Red stained sections highlighting the presence of congophilic inclusions (arrows). **e**, **f** Acid phosphatase over-reactivity within vacuoles (arrows) and in a punctuate fashion outside the vacuoles, suggesting lysosomal dysfunction. **g**, **h** Ectopic p62 immunoreactivity occurs focally (arrow) or diffusely (asterisk) in numerous muscle fibers. **a**–**h** images are ×40, scale bars: 20 µm.

Patient 1 (denoted as GNE^1^) was a 23-year-old Indian male with insidiously progressive bilateral foot dorsiflexor weakness since age 14. The weakness extended to proximal lower limb muscles and then to the upper limbs, first distally and then proximally. Neurological examination showed moderate to severe weakness in the lower limbs with plegia (Medical Research Council, MRC 0) of the leg muscles below the knee, hamstrings, and iliopsoas and less prominent involvement of quadriceps (MRC 4+) and glutei muscles (Medical Research Council, MRC 4−, 3.5), generalized severe upper limb muscle weakness (MRC 2, 3) with relative sparing on the thenar muscles (MRC 4). He was unable to ambulate independently. Creatine kinase (CK) was 581 U/L (normal 52–336). Electromyography (EMG) study showed diffuse myopathic changes with fibrillation potentials. A biopsy of the gluteus medius (MRC 4−; Fig. 1) showed a myopathy with rimmed vacuoles. There was an increased endomysial and perimysial connective tissue that replaced almost entirely part of the biopsy specimen. He was compound heterozygous for two missense GNE mutations, c.479 G > A (p.Arg160Gln) and c.2179 G > A (p.Val727Met).

Patient 2 (denoted as GNE^2^) was a 24-year-old White male who played football until age 16. He manifested distal lower limb weakness around age 19. The weakness extended to the proximal lower limb and then upper limb muscles. Neurological examination demonstrated moderate diffuse lower limb weakness (MRC 3, 4−) but normal quadriceps strength (MRC 5) and mild upper limb weakness (MRC 4, 4−). He had a waddling and stepping gait. CK was 1314 U/L. EMG findings showed diffuse myopathic changes with fibrillation potentials recorded in all tested muscles except for vastus lateralis and vastus medialis. Biopsy of the triceps (MRC 4; Fig. 1) also showed a myopathy with rimmed vacuoles. He was compound heterozygous for two GNE mutations, c.1835G > T (p.Cys612Phe) and c.2218 G > A (p.Ala740Thr).

We re-examined the diagnostic histopathological features of two GNEM patient biopsies by assessing standard GNE myopathy characteristics^1,18^ to establish a baseline for eventual comparison to patient-derived cells. We also performed immunohistochemical studies. First, hematoxylin and eosin (H&E), as well as modified Gomori trichrome staining performed during clinical testing, revealed that both GNEM patient samples, GNE^1^ and GNE^2^, contained myofibers of variable size (Fig. 1a, b). Second, both samples featured rimmed vacuoles, which were more abundant in GNE^1^ compared to GNE^2^ (Fig. 1a, b, arrows). On biopsy, GNE^1^ revealed myriad myofibers harboring one or more rimmed vacuoles, which were much less frequent in GNE^2^ and occurred with variable frequency in various areas of the specimen (about 8 vacuoles per lower power field, ×10, varying from 1 to 14). Congo Red staining revealed congophilic inclusions (Fig. 1c, d, arrows), while acid phosphatase staining showed reactivity, especially in the vacuoles (Fig. 1e, f, arrows). Autophagy pathway activity was queried by staining muscle cross-sections for p62 reactivity^19,20^. Here, two distinct patterns of p62 were observed: focal accumulation of p62 (arrows) and diffuse p62 staining throughout the myofiber (asterisk) (Fig. 1g, h). Both genetically confirmed GNEM patient muscle biopsies exhibited the histopathological GNEM hallmarks. The more severe clinicopathological findings in GNE^1^ support the impression that compound heterozygous GNE myopathy patients with one mutation in each domain of GNE may have a more severe phenotype compared to those harboring both mutations in either the epimerase or kinase domain^21^. However, phenotypic variability can occur in the setting of the same genotype. In addition, one should keep into account the different sites of biopsy in the two patients and the variable distribution of structural changes across muscle tissue that can be observed in various inherited myopathies.

### Patient-derived induced pluripotent stem cells (iPSCs) exhibit GNE myopathy hallmarks and differentiate poorly into mature skeletal muscle myotubes

**Fig. 2:**
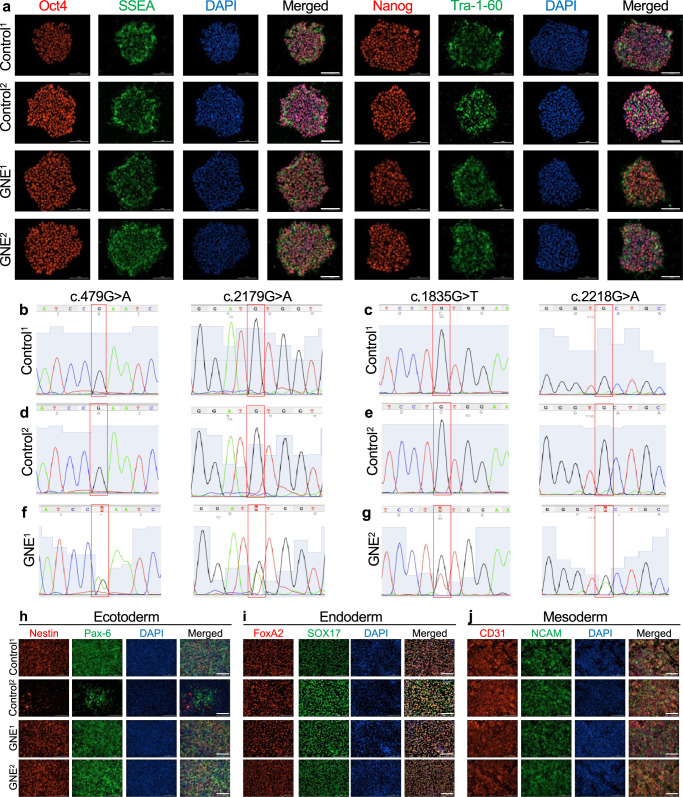
iPSC confirmation of pluripotency marker expression, GNE mutation sequencing, and germ layer differentiation capabilities. **a** Control^1^ (top row), Control^2^ (second row), GNE^1^ (third row), and GNE^2^ (bottom row) iPSCs were assessed for expression of standard pluripotency markers by immunofluorescence (IF). All clones expressed Oct4, SSEA, Nanog, and Tra-1-60. **b**–**g** Sanger sequencing plots confirming the presence of the compound heterozygous patient mutations. Control samples contain the wild-type alleles at all mutant locations (red boxes, Control^1^: **b**, **c**, Control^2^: **d**, **e**), whereas GNE^1^ are heterozygous for wild-type and mutant alleles (red boxes, **f**) as is GNE^2^ (red boxes, **g**). **h**–**j** IF images highlighting germline differentiation capacity. Differentiated iPSCs were capable of expressing standard ectoderm (Nestin and Pax-6) (**h**), endoderm (FoxA2 and SOX17) (**i**), and mesoderm (CD31 and NCAM) markers (**j**). All images are representative. **a** and **h**–**j** images are ×20, scale bars: 100 μm.

Punch biopsies from both patients' GNEM were collected, and fibroblast cultures were established. Healthy (no myopathy) controls and GNEM patient-derived fibroblasts were reprogrammed into induced pluripotent stem cells (iPSCs) using Sendai virus-Cytotune 2.0. Two clones were derived from the healthy control lines (Control^1^ and Control^2^), and three clones derived from each of the two GNEM patient lines (GNE^1^ and GNE^2^, Table 1). In addition, for assessment of differentiation in another hereditary myopathy and a male origin control patient, one clone from an additional third healthy control line (Control^3^) and one clone from a patient with nemaline myopathy (ACTA1) were also assessed (Supplementary Table 1). All clones underwent rigorous quality control characterization routinely performed in the iPSC field. In brief, iPSC identity was confirmed by short tandem repeat (STR) analysis, found negative for mycoplasma, and exhibited normal karyotypes via G-banding. Pluripotency markers were assessed, and all clones were positive for Oct4, SSEA, Nanog, and TRA-1-60 (Fig. 2a and Supplementary Fig. 1A), with no gross abnormalities apparent. Since it has been shown that the iPSC reprogramming process can itself introduce genetic variation/drift^22^, we utilized Sanger Sequencing to confirm the retention of *GNE* mutations post-reprogramming in one set of clones from each GNEM patient line and control line 1 and control line 2 (Control^1^ and Control^2^, respectively). The two control lines demonstrated homozygous genotypes containing wild-type alleles, seen as the presence of a single nucleotide peak (Fig. 2b–e, red boxes). Conversely, GNE^1^ contained double peaks of relatively equal size for both G and A at c.479 and c.2179 (Fig. 2f, red boxes), indicating the presence of a single mutation in both the epimerase and kinase domains. GNE^2^ contained double peaks for G and T at *GNE* c.1835 and G and A at c.2218, respectively (Fig. 2g, red boxes), which corresponds to two mutations in the kinase domain of GNE. These Sanger Sequencing results matched the original patient compound heterozygous mutations found at diagnosis (Table 1). To confirm germline differentiation capacity, all clones were tested for proficiency to differentiate into all three germ layers. Standard ectoderm, endoderm, and mesoderm markers were qualitatively assessed post-differentiation (Fig. 2h–j and Supplementary Fig. 1B–D). All control, GNEM, and ACTA1 patient-derived iPSC clones were capable of expressing Nestin and Pax-6 post-ectoderm differentiation (Fig. 2h and Supplementary Fig. 1B), FoxA2 and Sox17 after endoderm differentiation (Fig. 2i and Supplementary Fig. 1C), and CD31 and NCAM for cells differentiated down a mesoderm lineage (Fig. 2j and Supplementary Fig. 1D). These results demonstrate successful reprogramming of patient-derived fibroblasts into functional iPSCs.

**Table 1:** Features of two healthy or GNEM patients of which iPSC lines were derived.

Line	Number of clones	Mutations in GNE	Sex	Age (years)
Control^1^	2	N/A	Female	37
Control^2^	2	N/A	Female	53
GNE^1^	3	c.479 G > A (p.Arg160Gln) c.2179 G > A (p.Val727Met)	Male	23
GNE^2^	3	c.1835G > T (p.Cys612Phe) c.2218 G > A (p.Ala740Thr)	Male	24

Controls, GNE^1^, and GNE^2^ are listed by name and subsequent number of clones per line, presence of GNE mutations, sex, and age are described.

*N/A* not applicable.

**Fig. 3:**
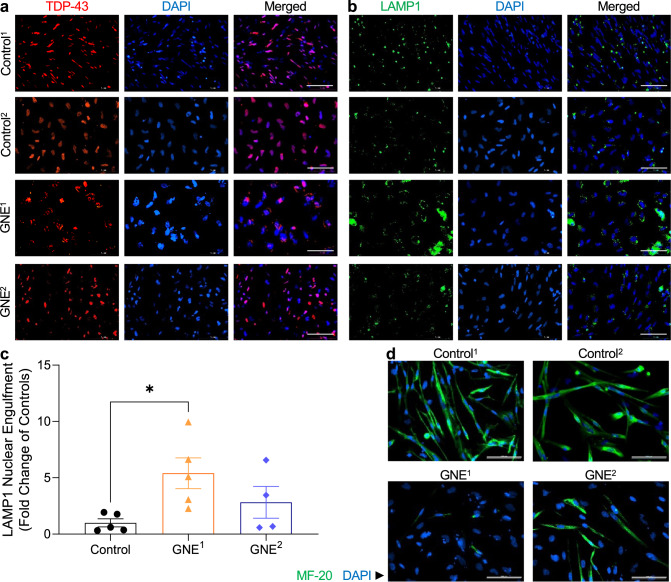
Recapitulation of human GNEM characteristics in GNEM patient-derived iPSC lines. **a**, **b**, **d** IF images depicting GNE markers and myosin heavy chain expression. Control^1^, Control^2^, GNE^1^, and GNE^2^ iPSCs were differentiated to myotubes and stained for **a** standard GNE markers including TDP-43 (red) and DAPI (blue) or **b** LAMP1 (green) and DAPI (blue) and **d** myosin heavy chain (MF-20, green) and DAPI (blue). **c** Quantification of LAMP1 nuclear engulfment. All images are representative; **a**, **b** and **d** were taken at ×20, scale bars: 100 µm. Each point in quantification represents *n* = 1 from the average values of 4–5 images per well, *n* = 4–5. **c** = Student’s unpaired *t*-test where data are presented as mean ± standard error of the mean (SEM). Significance is *p < 0.05, **p < 0.01, ***p < 0.001.

Next, we sought to determine the extent to which GNEM patient-derived cells exhibited characteristics of GNE myopathy in vitro. Expression patterns of TAR DNA Binding Protein 43 (TDP-43) and LAMP1 were assessed in control and GNEM patient-derived cells subjected to a 3-step myogenic differentiation protocol to form skeletal muscle myotubes. TDP-43 is known to be present in the protein aggregates of GNE myopathy^23^ as well as having ectopic sarcoplasmic expression in other myopathies with rimmed vacuoles, including sporadic inclusion body myositis^24–26^. In control-derived myotubes, TDP-43 signal colocalized with the nuclear stain, 4’,6-diamidino-2-phenylindole (DAPI), as expected (Fig. 3a and Supplementary Fig. 2A). Strikingly, GNE^1^ myotubes exhibited TDP-43 signal in some cytoplasmic regions (Fig. 3a), with some normal TDP-43 and DAPI overlap. GNE^1^ also exhibited increased LAMP1, a lysosomal marker suggesting the presence of autophagy^27^, nuclear engulfment compared to controls (Fig. 3b, c and Supplementary Fig. 2A). Alternatively, GNE^2^ myotubes appeared to have an intermediate phenotype between controls and GNE^1^ regarding expression patterns of TDP-43 and LAMP1 (Fig. 3a–c and Supplementary Fig. 2A). Finally, compared to controls and the ACTA1 samples, we observed almost no MYH1 (using the monoclonal antibody MF-20) reactivity in GNE^1^ and reduced expression in GNE^2^ (Fig. 3d and Supplementary Fig. 2B), suggesting that myogenic differentiation is impaired in GNE myopathy iPSCs, but importantly is not the case in all myopathy-derived iPSCs, progressing down a skeletal muscle lineage.

### Patient-derived induced pluripotent stem cells (iPSCs) exhibit alterations in myogenic regulatory factor (MRF) expression during myogenic differentiation

**Fig. 4:**
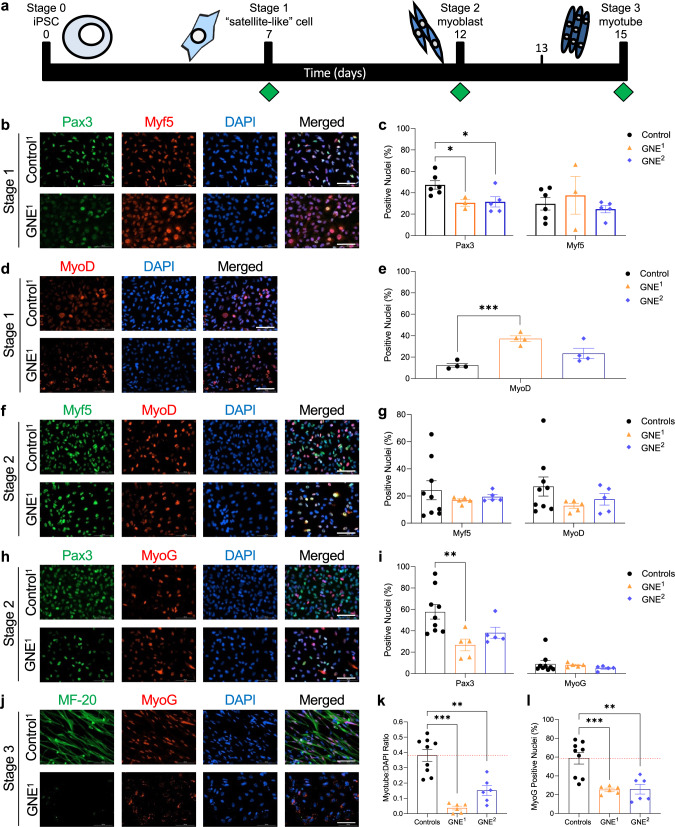
Muscle regulatory factor (MRF) expression during myogenic progression of healthy and GNEM patient-derived iPSCs. **a** A schematic depicting the experimental timeline. Green diamonds represent the time of sample preparation for immunofluorescence. **b**–**e** Satellite-like cell, or stage 1 iMPCs. MRF protein expression in Control^1^ (top) and GNE^1^ (bottom) cells stained with **b** Pax3 (green), Myf5 (red), and DAPI (blue) or **d** MyoD (red) and DAPI (blue). **c**, **e** Quantification of the percent positive nuclei from controls (Control^1^ and Control^2^ combined, black), GNE^1^ (orange), and GNE^2^ (blue) in images **b** and **d**, respectively. **f**–**i** Protein expression of myogenic transcription factors of stage 2 iMPCs. **f**, **h** Control^1^ (top) and GNE^1^ (bottom) stage 2 cells stained with **f** Myf5 (green), MyoD (red), and DAPI (blue) and **h** Pax3 (green), MyoG (red), and DAPI (blue). **g**, **i** Quantification of the percent positive nuclei from controls (Control^1^ and Control^2^ combined, black), GNE^1^ (orange), and GNE^2^ (blue) in images **f** and **h**, respectively. **j**–**l** Control^1^ (top) and GNE^1^ (bottom) iMPCs were differentiated to myotubes (stage 3) and stained for myogenic markers MyoG (red) and myosin heavy chain (green). **k** Quantification of number of myotubes to DAPI ratio per ×20 image. **l** Quantification of the percent positive nuclei from controls (Control^1^ and Control^2^ combined, black), GNE^1^ (orange), and GNE^2^ (blue). All images are ×20 representative images, scale bar: 100 µm. Each point in quantifications represents *n* = 1 from the average values of 4–5 images per well, *n* = 3–9. All statistical analyses were performed using Student’s unpaired *t*-test and data are presented as mean ± SEM. Significance is *p < 0.05, **p < 0.01, ***p < 0.001.

Given the striking inability of GNE^1^ iPSCs to form mature skeletal muscle myotubes, we next sought to query stage-specific myogenic regulatory factor (MRF) protein expression in differentiating control, GNEM, and nemaline myopathy iPSCs (hereby referred to as induced myogenic progenitor cells, or iMPCs). Three iMPC stages were assessed that correspond to early, intermediate, and late stages of myogenesis: (1) stage 1- satellite-like cells, (2) stage 2- myoblasts, and (3) stage 3- myocytes/myotubes (Fig. 4a, green diamonds). Immunofluorescence was performed on two clones from Control^1^ and Control^2^, three clones from both GNE myopathy patient-derived lines and one clone from each Control^3^ and ACTA1 patient-derived lines. Nuclear-overlapping MRF expression was quantified as a percentage of total nuclei. At stage 1, both GNE^1^ and GNE^2^-derived samples exhibited a statistically significant decrease (16.80 ± 6.19% and 15.79 ± 6.20%, respectively) of Pax3 percent positive nuclei when compared to controls (combined quantification of Control^1^ and Control^2^), while no difference was observed in Myf5 positive nuclei (Fig. 4b, c and Supplementary Fig. 3A). GNE^1^ displayed an increased fraction of MyoD positive nuclei compared to control (24.81 ± 3.22% (*p* = 0.0002)), whereas GNE^2^ was trending (11.12 ± 5.06% (*p* = 0.070)) (Fig. 4d, e and Supplementary Fig. 3B). No differences in Myf5, MyoD, or MyoG positive nuclei were observed in stage 2 cells between GNEM or iMPC controls (Fig. 4f–i and Supplementary Fig. 3C, D). A statistically significant reduction in the fraction of Pax3 positive nuclei persisted in GNE^1^, but not GNE^2^ samples, at stage 2 compared to controls (31.00 ± 10.10% (*p* = 0.0097) and 19.65 ± 10.02% (*p* = 0.074), respectively) (Fig. 4h, i and Supplementary Fig. 3D). Additionally, both Control^3^ and ACTA1 cells similarly express these markers across all differentiation time points (Supplementary Fig. 2E–G). At stage 3, MF-20 was abundantly detected in control myotubes, with very little to no expression found in the iMPC sample derived from GNE^1^ iPSCs (Fig. 4j, Supplementary Fig. 2B, and Supplementary Fig. 3E). The ratio of myotubes to nuclear signal was calculated and both GNE^1^ and GNE^2^ samples had significantly decreased myotube counts compared to controls (34.41 ± 4.91% and 22.97 ± 5.47%, respectively) (Fig. 4k). The male origin control (Control^3^) and the ACTA1-derived patient cells could differentiate into myotubes at a similar level as Control^1^ and Control^2^ (Supplementary Fig. 2C, dashed red line is controls average from 4 K). In addition, MyoG extensively overlapped with DAPI in control and ACTA1 myotubes (Fig. 4j, Supplementary Fig. 2B, and Supplementary Fig. 3E) but was rarely detected as an overlap in GNE^1^ and less in GNE^2^ (Fig. 4j and Supplementary Fig. 3E). This is reflected in the quantification of MyoG percent positive nuclei where controls had significantly increased percentage of MyoG percent positive nuclei compared to both GNE^1^ and GNE^2^ (33.42 ± 7.75% and 32.95 ± 8.68%, respectively) (Fig. 4l). Quantification of Control^3^ and ACTA1 also revealed similar levels of MyoG percent positive nuclei as Control^1^ and Control^2^ (Supplementary Fig. 2D, dashed red line is controls average from 4l).

### Widespread global transcriptomic differences between healthy and GNEM patient-derived iPSC/iMPC samples during myogenic differentiation

**Fig. 5:**
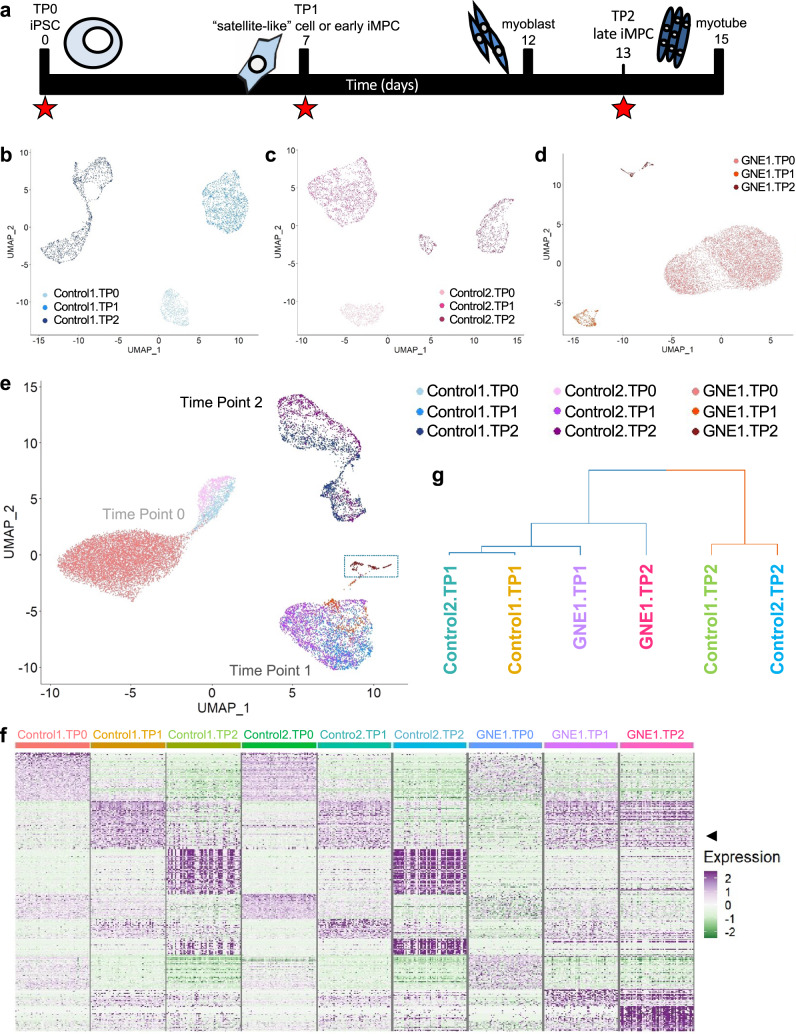
Global transcriptome differences between healthy and GNEM patient-derived iPSCs undergoing myogenic differentiation. **a** A schematic depicting the experimental timeline. Red stars correspond to collection of samples for single-cell RNA sequencing; first star: Time Point 0; second star: Time Point 1 or early iMPCs; third star: Time Point 2 or late iMPCs. **b**–**e** UMAP projection of single-cell data from **b** Control^1^ (Control1), **c** Control^2^ (Control2), **d** GNE^1^ (GNE1), and **d** Control1, Control2, and GNE1 combined, containing Time Points 0, 1, and 2. **f** Heatmap depicting the log scale of the top 100 DEGs, as represented by 200 cells from each group, for Control1, Control2, and GNE1 Time Points 0, 1, and 2. Purple: higher expression and green: lower expression. **g** Hierarchical clustering depicted via dendrogram using the log scale average of the top 100 DEGs from Control1, Control2, and GNE1 Time Points 1 and 2. Clustering method: complete linkage. Distance measure: correlation.

Next, we sought to query molecular (transcriptional) alterations in differentiating control and GNEM patient iPSCs/iMPCs. We selected three time points, iPSC (time point 0), satellite-like cells or early iMPCs (time point 1), and shortly after myoblast formation but not yet myotubes, or late iMPCs (time point 2) for these studies (Fig. 5a, red stars). Single cells were prepared using a 10× Genomics workflow and sequenced on an Illumina HiSeq4000 platform. Quality control (QC) was performed using Illumina software and the Seurat package in R (Supplementary Table 2). Nine samples in total were analyzed, containing one clone from Control^1^, one clone from Control^2^, one clone from GNE^1^ (these specific clones are depicted in Figs. 3, 4, and Supplementary Fig. 3), and one clone from ACTA1 (clone depicted in Supplementary Fig. 2). Control^1^ and Control^2^ samples from each time point 0 (Control1 or 2.TP0), 1 (Control1 or 2.TP1), and 2 (Control1 or 2.TP2) were combined and visualized individually by the Uniform Manifold Approximation and Projection (UMAP) (Fig. 5b, c). The control samples demonstrate distinct clustering of each cell stage, indicating relatively dissimilar transcriptomic profiles across differentiation, as expected. This was also seen when ACTA1 cells were plotted via UMAP at time point 0 (ACTA1.TP0), 1 (ACTA1.TP2), and 3 (ACTA1.TP3) (Supplementary Fig. 4A). GNE^1^ cells at time points 0 (GNE1.TP0), 1 (GNE1.TP1), and 2 (GNE1.TP2) were similarly visualized by UMAP and exhibited distinct stage-specific clustering (Fig. 5d). We combined controls (Control^1^ and Control^2^) and GNE1 samples in a single UMAP plot (Fig. 5e), or also including ACTA1 samples (Supplementary Fig. 4B), and observed that control and GNE1 samples clustered reasonably well together at TP0 and TP1. This was also true when the ACTA1 samples are included. Strikingly, GNE1.TP2 iMPCs clustered just outside of Control1/Control2/(ACTA1)/GNE1.TP1 samples, whereas Control^1^/Control^2^/(ACTA1).TP2 samples clustered distantly in the upper right (Fig. 5e) or left quadrant (Supplementary Fig. 4B).

We next visualized the top 100 differentially expressed genes (DEGs) between Control^1^, Control^2^, and GNE^1^ TP0, TP1, and TP2 in a heatmap containing 200 random cells from each of these time points (Fig. 5f), as well as with ACTA1 (Supplementary Fig. 4C). When comparing Control^1^, Control^2^, (ACTA1), and GNE^1^ TP1 and 2, we found that the Controls/(ACTA1)/GNE1.TP1 samples were most similar, whereas Control^1^/Control^2^/(ACTA1).TP2 exhibited clear qualitative differences in transcript expression. In contrast to Control^1^/Control^2^/(ACTA1).TP2, GNE1.TP2 appeared more similar to Controls/(ACTA1)/GNE.TP1. Notably, we observed a cluster of transcripts (Fig. 5f dark purple, bottom right and Supplementary Fig. 4C brown, bottom right) that characterized GNE1.TP2, with little to no expression observed in any other sample. Hierarchical clustering was performed on the log-normalized average gene expression values from the same 100 DEGs per sample from Control^1^/Control^2^/GNE^1^ TP1 and 2. Clustering clearly revealed the separation of Control1 and Control2.TP2 from the four other samples, Control1.TP1, Control2.TP1, GNE1.TP1, and GNE1.TP2 (Fig. 5g). Markedly, the trend in the similarity between GNE1.TP1, GNE1.TP2, Control1.TP1 and Control2.TP1 continued to be present when viewing the top five DEGs per sample at TP1 and 2 (Supplementary Fig. 5).

**Fig. 6:**
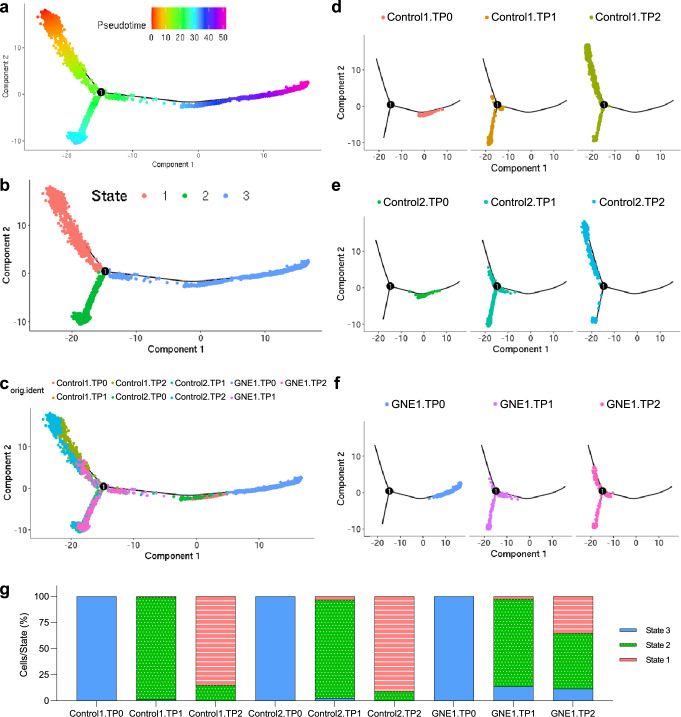
Pseudotime analysis reiterates inhibited myogenic progression of GNEM patient-derived cells compared to healthy controls. **a**–**f** Pseudotime trajectory analysis across all time points using single cells from Control^1^ (Control1), Control^2^ (Control2), and GNE^1^ (GNE1). **a** Single-cell projected trajectory painted by pseudotime across all differentiation states, **b** the 3 projected state branches were based on differentially expressed genes (DEGs), **c** all single-cell samples mapped onto branches, and **d** Control1, **e** Control2, and **f** GNE1 individual single-cell samples mapped onto branches. **g** Proportion of cells from each sample that fall within state 1 (red and lined), state 2 (green and dotted), and/or state 3 (blue solid).

We then performed a pseudotime trajectory analysis (Monocle) to better capture the stage transition defects observed in GNE1.TP2 cells. First, we used pseudotime to predict the chronological order of cell differentiation (Fig. 6a) and for the categorization of cell states (Fig. 6b). These analyses revealed three distinct cell states (right, upper, lower trajectories), corresponding to the three stages of myogenic differentiation assessed (Fig. 6b). The beginning of ordered pseudotime prediction was state 3 (Fig. 6a, b—right branch) which is predominately made up of Control1.TP0, Control2.TP0, and GNE1.TP0 (Fig. 6b–f), the most primitive state prior to induction of myogenesis. The initial branch, state 3, then split into two divergent branches (Fig. 6b), where branch 2 was predicted to be more mid-late differentiation and contained the vast amount of Control1.TP1, Control2.TP1 and GNE1.TP1 cells (Fig. 6b–f—lower branch). These cells also populated a segment close to the divergence point of branch 1 (Fig. 6b–f—upper branch), whereby branch 1 was predicted to be the mid-late to late-stage differentiated cells (Fig. 6a). Control1.TP2 and Control2.TP2 displayed a few cell clusters on the state 2 branch but are largely distributed along the upper extended state 1 branch. Contrary to Control1.TP2 and Control2.TP2 cell disbursement (Fig. 6b–e), GNE1.TP2 exhibited a trajectory pattern more similar to that of Control1.TP1 and Control2.TP1 cells such that GNE1.TP2 did not display robust extension on the upper state 1 branch (Fig. 6b, c, and f).

We further validated the projected chronological ordering of pseudotime by assessing MRF expression across the states during the projected pseudotime ordering (Supplementary Fig. 6). PAX3 expression showed little to no expression at the earliest time point, state 3, and increased in states 2 and 1, indicating the differentiation from iPSC to iMPCs (Supplementary Fig. 6B). Similarly, expression patterns of late-stage MRFs, like MYOD, MYOG, and DES, were predominately expressed in stage 1 cells (Supplementary Fig. 6D–F), confirming the end stage differentiation status of cells residing along this trajectory. Finally, sample quantification of the percentage of cells that fell into each state (Fig. 6g) also suggested altered myogenic progression of the GNE sample. In Control1.TP1 and Control2.TP1, 98.30% and 94.36% of cells in these samples fell into state 2 cells, whereas only 0.62% and 3.39% fell on the state 1 branch, respectively (Fig. 6g). Comparatively, GNE1.TP1 cells had 83.76% and 2.58% fall into state 2 and 1, respectively (Fig. 6g). Notably, Control1.TP2 cells were 14.97% state 2 and 84.97% state 1, Control2.TP2 cells were 9.06% state 2 and 90.94% state 1, whereas GNE1.TP2 were 53.23% state 2 and 35.32% state 1 (Fig. 6g). In addition, when pseudotime was performed including ACTA1 cells, the most late-stage differentiation branch, state 1, contained 87.64% of Control1.TP2, 91.62% Control2.TP2, 77.16% ACTA1.TP2, but only 35.82% GNE1.TP2 cells (Supplementary Fig. 4D). Collectively, these data provide strong molecular evidence in support of the poor observed myogenic differentiation of the GNE1.TP2 compared to all other Controls and even ACTA1.

### Alteration of key myogenic regulatory factor transcripts and the elucidation of a contributing mechanism to myogenic dysfunction in GNEM iPMCs

These single-cell data highlight significant transcriptional changes between GNE and control cells at several time points, but most notably time point 2. Given that GNE is the rate-limiting step in sialic acid biosynthesis, we next sought to determine whether sialic acid levels were altered at these time points. Sialic acid levels were assessed at stage 2 (equivalent to single-cell TP1) and stage 3 (more differentiated than single-cell TP2, contains myotubes) cells using a biotinylated Sambucus Nigra Lectin (SNA) antibody and western blotting (Supplementary Fig. 7A, B). SNA signal was normalized to total protein, and no differences were seen between controls (quantification includes Control^1^ and Control^2^) and either GNE patient-derived samples at stage 2 or stage 3 (Supplementary Fig. 7C, D).

**Fig. 7:**
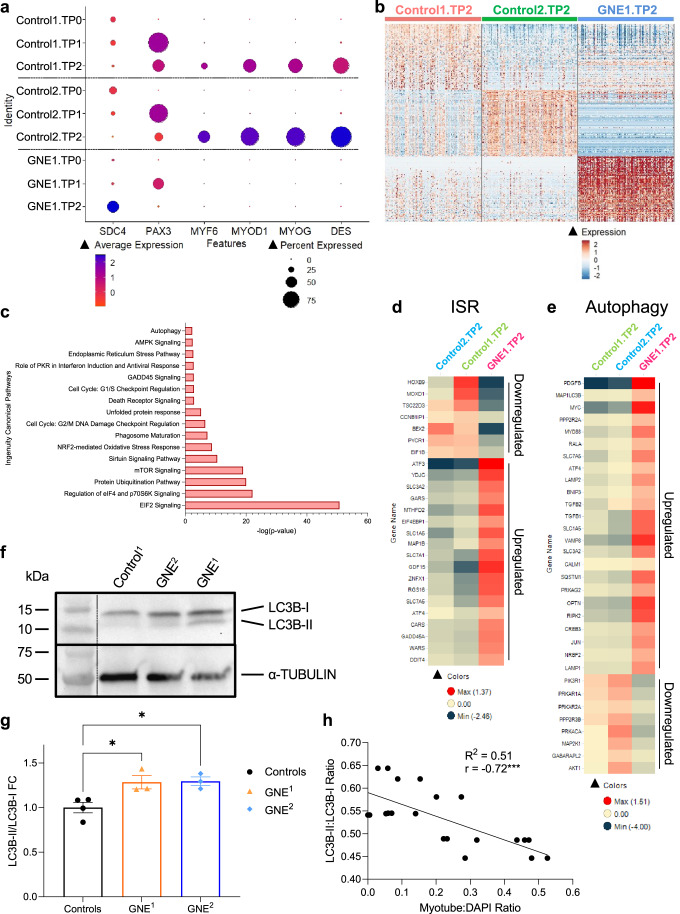
Identification of altered stress-related signaling pathways and autophagy in GNE myopathy iMPCs. **a** Dot plots of selected MRFs in Control^1^ (Control1), Control^2^ (Control2), and GNE^1^ (GNE1) samples at Time Points 0, 1, and 2. Blue: higher expression, red: lower expression. **b** Heatmap illustrating all DEGs between Control1, Control2, and GNE1 Time Point 2 (Control1.TP2, Control2.TP2, and GNE1.TP2, respectively). Red: high expression and blue: low expression. **c** Ingenuity pathway analysis (IPA) results using all DEGs found between Control1, Control2, and GNE1 Time Point 2 (shown in **b**), whereby represented pathways were significantly altered Ingenuity Canonical Pathways in relation to stress and autophagy. **d**, **e** Heatmaps depicting average transcript expression of significantly altered integrated stress response (ISR, **d**) or autophagy-related (**e**) genes across Control1, Control2, and GNE1 at TP2. **f** Representative western blot from Control^1^, GNE^2^, and GNE^1^ Stage 3 samples probed for LC3B and α-tubulin. Ladder was stitched to blot, see original blot in Supplementary Fig. 11A. **g** Quantification of Stage 3 LC3B-II/LC3B-I ratios in controls (combined Control^1^ and Control^2^), GNE^1^, and GNE^2^. **h** Correlation analysis of LC3B-II/LC3B-I ratio versus myotube:DAPI ratio (values from 7 G and 4 K, respectively). **g** = Student’s unpaired *t*-test, *n* = 3–4, and data are presented as mean ± SEM. **h** = Pearson’s correlation analysis. Significance is *p < 0.05, **p < 0.01, ***p < 0.001.

Assessment and visualization of myogenic transcript/MRF expression across time points revealed several striking differences between Control^1^, Control^2^, ACTA1, and GNE^1^ samples (Fig. 7a, Supplementary Fig. 4E, and Supplementary Fig. 8). First, PAX3 transcript expression in both Control1 and Control2.TP1 samples was (a) detected in the vast majority of cells, and (b) expressed at a comparatively high level. At GNE1.TP1, there was a reduced number of PAX3-expressing cells and a reduced level of expression per cell (Fig. 7a and Supplementary Fig. 8B). Second, controls at TP2 exhibited an increase in the number of cells and intensity of expression of MYOD, MYOG, and DES compared to controls at TP1. This effect was not observed in GNE1.TP2 samples (Fig. 7a and Supplementary Fig. 8D–F). Additionally, we performed quantitative real-time PCR (qRT-PCR) for MRFs on all clones from Control^1^, Control^2^, GNE^1^, and GNE^2^ samples at stages 1, 2, and 3 (Supplementary Fig. 8G–I). In accordance with previous data, stage 2 control cells exhibited trends towards increased MyoG, a late-stage MRF (Supplementary Fig. 8H) and lower expression of early MRFs (Pax3, Myf5, and MyoD) at stage 3 (Supplementary Fig. 8I).

To address underlying mechanisms that could be contributing to an impaired GNE1.TP1 to TP2 transition, we identified DEGs between GNE1.TP2, Control1.TP2, and Control2.TP2 iMPCs (Fig. 7b) and performed ingenuity pathway analyses (IPA, Supplementary Table 3). IPA identified numerous aberrantly activated stress-related signaling pathways (e.g., EIF2 signaling, unfolded protein response, endoplasmic reticulum stress pathway, etc) and autophagy-related pathways (e.g., protein ubiquitination pathway, phagosome maturation, and autophagy). Although not all mentioned pathways are represented in the top 25 altered IPA pathways (Supplementary Table 3), they are all significantly altered in GNE1.TP2 compared to Control1.TP2 and Control2.TP2 (Fig. 7c–e). Further, we recognize that although there are many pathways more significantly over-represented with respect to “autophagy”, many of these pathways directly relate to autophagic flux and are characterized by transcripts known to be associated with autophagy. Thus, to query potential dysfunctional autophagy signaling in GNE cells, we evaluated microtubule-associated proteins 1 A/1B light chain 3B (LC3B) levels in stage 3 control and GNE cells. As cleaved LC3B (LC3B-II) is indicative of autophagosome presence (a key intermediate autophagy factor), the ratio between LC3B-II and LC3B-I (uncleaved LC3B) was assessed via western blot (Fig. 7f) to query relative autophagy status. The ratio of LC3B-II/LC3B-I was significantly increased in GNE^1^ and GNE^2^ samples compared to Controls (Control^1^ and Control^2^) at stage 3, or late-stage differentiation (Fig. 7g). Additionally, correlation analysis was performed using myotube:DAPI ratio (from Fig. 4k) versus the LC3B-II/LC3B-I ratio. Pearson’s correlation revealed a significant negative correlation between myogenic potential/capacity and LC3B cleavage (Fig. 7h). Conversely, when myotube:DAPI ratio was plotted versus stage 3 SNA levels, no correlation was found (Supplementary Fig. 9).

**Fig. 8:**
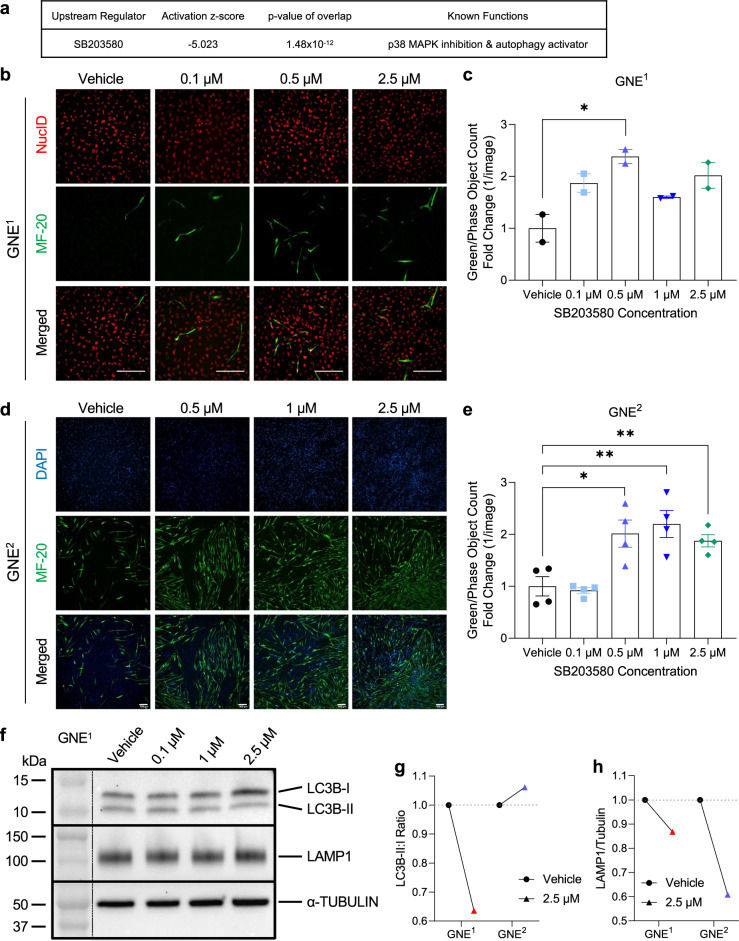
Improved GNEM iMPCs myotube formation following SB203580 treatment. **a** IPA upstream regulator analyses identified SB203580 as a candidate compound able to mitigate transcriptional differences observed between GNE.TP2 and Control (Control1.TP2 and Control2.TP2) cells. **b**–**e** IF images and quantification of GNE^1^ and GNE^2^ cells treated with vehicle or varying concentrations of SB203580. GNE cells were differentiated to myotubes and treated with SB203580 for remainder of differentiation after cell adherence during stage 2. **b**, **d** IF images depicting Nuclear ID (red, **b**) or DAPI (blue, **d**) and MF-20 (green) from GNE^1^ vehicle, 0.1 μM, 0.5 μM, and 2.5 SB203580 treated cells (**b**) or GNE^2^ vehicle, 0.5 μM, 1 μM, and 2.5 μM SB203580 treated cells (**d**). **c**, **e** Quantification depicts the fold change in the number of total green objects per phase objects, per ×10 image. **f** Representative western blot of vehicle, 0.1, 1, and 2.5 μM SB203580 treated GNE^1^ cells probed for LAMP1, LC3B, and α-tubulin. Ladder was stitched to blot, see original blot in Supplementary Fig. 11E. **g**, **h** Quantification of LC3B-II/LC3B-I (**g**) or LAMP1 (**h**) fold change from 2.5 μM SB203580 treated cells versus vehicle. All images are representative. **b** = ×20 with scale bars: 200 µm. **d** = ×10, scale bars: 100 µm. Each point in quantification represents *n* = 1 and is the average values of 16 or 36 images per well, *n* = 2–4. Statistical analysis was performed using Student’s unpaired *t*-test and data are presented as mean ± SEM. Significance is *p < 0.05, **p < 0.01, ***p < 0.001.

Upstream regulator analysis of stage 3 DEGs identified SB203580 as a candidate compound for possible mitigation of GNE-associated signaling defects (Fig. 8a). We therefore aimed to determine if SB203580 treatment could improve GNEM-associated myogenesis defects. The clone with the most severely affected myogenic capacity from each GNE^1^ and GNE^2^ iPSCs was differentiated and treated with either vehicle or varying concentrations of SB203580 starting at stage 2 cell adherence and continuing through the experimental endpoint. Terminally differentiated cells were stained with MF-20 and analyzed (Incucyte) to quantify green (MF-20) and phase counts per image (GNE^1^: Fig. 8b, c and GNE^2^: Fig. 8d, e). Green object count was normalized to total phase objects per image to account for any cell density variability between treatments. Fold-change calculations were compared to vehicle-treated cells (GNE^1^: Fig. 8c and GNE^2^: Fig. 8e). GNE^1^ and GNE^2^ cells exhibited an increase in myogenic differentiation capacity when treated with SB203580 (Fig. 8b–e).

As SB203580 is a well-known p38 MAPK inhibitor, we wanted to assess whether the alteration of this pathway could be partly responsible for the decreased ability of the GNEM samples to differentiate into myotubes. The activation status of the p38 pathway (p-p38/p38 ratio) was assessed in stage 2 and stage 3 samples via western blot (Supplementary Fig. 10A–D). Quantification demonstrated that the activation status of the p38 signaling pathway was not altered in GNE^1^ or GNE^2^ in stage 2 or 3 differentiated cells (Supplementary Fig. 10B and Supplementary Fig. 10D). To confirm this result, the activation status of MK2, a known downstream target of p38 was also determined in stage 3 cells (Supplementary Fig. 10E, F). Again, no difference in the activation status of MK2 was seen (Supplementary Fig. 10F). SB203580 is also reported to induce autophagy/enhance autophagic flux^28^. Our previous data demonstrated an increase in the ratio of LC3B-II/LC3B-I in GNEM patient-derived cells, so we determined whether SB203580 had any impact on this ratio as well as on the amount of LAMP1 (another autophagy component) via western blot (Fig. 8f). SB203580 decreased either, or both, LC3B-II/LC3B-I and LAMP1 levels in the clones that demonstrated improved myogenic outcomes (Fig. 8g, h), suggesting the drug may be imparting its beneficial effects by enhancing autophagy activation and/or relieving a block in autophagic flux in GNEM patient-derived cells.

## Discussion

This study describes the generation of a patient-derived, cell-based GNEM model that provides numerous advantages over existing GNEM model systems. First, the utilization of two GNEM patient-derived samples (Figs. 1, 2) permits the modeling of diverse disease states and may facilitate genotype-to-phenotype studies not easily performed using mouse models (Figs. 3, 4). Second, as our GNEM model is cell-based and patient-derived, there is robust scaling potential for drug screening and/or therapeutic efficacy studies that are labor intensive and technically challenging using animal models. Third, patient-derived samples are relatively easy to obtain in a noninvasive way, and the reprogramming of iPSCs is now a standard and cost-effective platform. Fourth, iPSC-based models permit the maintenance of a patient’s entire genetic identity. Gene-by-gene and gene-by-environment interactions are critical when trying to understand the variable expressivity of disease-causative primary mutations and their contribution to clinical phenotype and disease mechanism. For example, inherited myopathies, including GNEM, can manifest with intrafamilial variability in regard to clinical phenotype, age of onset, or disease progression^29–31^. In addition, there is evidence of genetic modifiers accompanying the primary mutation or modulating the phenotype^32,33^. Therefore, a model system able to capture gene variability while also recapitulating the key pathological characteristics becomes crucial for elucidating yet-to-be discovered contributors to disease etiology.

Although there are many benefits associated with cell-based models, there is one drawback that cannot be overlooked. Extensive and accurate electrophysiological muscle studies, such as muscle force measurements, cannot be conducted using cells, thereby making animal models superior to cell-based models in this regard. To overcome this obstacle, there are efforts currently underway to introduce differentiated human iPSCs into mice for in vivo physiological assessments. For example, a new model to study Alzheimer’s disease (AD) was created, where normal human embryonic stem cells or iPSCs derived from a patient with fronto-temporal dementia were differentiated into neural precursors and implanted into mice to study the etiology of human AD in vivo^34^. Another example from Espuny-Camacho and colleagues found that cortical neurons, differentiated from human embryonic stem cells and implanted into the mouse brain, were able to produce human cerebral cortex-like characteristics in vivo^35^. Finally, in the third study by Harada et al., normal human iPSCs were differentiated into mesenchymal stromal cells (MSCs) and transplanted into a mouse model of Collagen VI-related myopathies (COL6A1^KO^) as a potential therapeutic strategy^36^. The authors found that COL6A1^KO^ mice with intraperitoneal injection of MSCs demonstrated COL6 expression in both the quadricep and diaphragm, increased TA and GR muscle weight, and improved grip strength, muscle force, and running time on rotarod when compared to COL6A1^KO^ ^36^. Such studies highlight the relevance and potential of transplant-based approaches to study human disease and therapeutics in animals. Indeed, we believe that the healthy and GNEM patient-derived iMPCs described herein are well-suited for these types of in vivo applications.

To our surprise, our single-cell RNA sequencing data revealed little to no expression of MyoD and MyoG transcripts in patient GNEM cells at all time points (Fig. 7), which on the surface, seems at odds with the immunofluorescence data reported during GNEM iPSC differentiation (Fig. 4). When probed by qRT-PCR, heterogeneity was still evident in some samples between clones of the same line, and within biological replicates. These data suggest the timing of sample collection could be very important for determining transcript/protein levels. This is supported by growing literature in the satellite cell field, which is that MRFs are subject to substantial post-transcriptional and post-translational modification^37–43^. For example, Crist and colleagues identified a pathway by which Myf5 mRNA is sequestered to mRNA/protein granules (RNPs) and is translationally “paused” with the help of microRNA-31, in quiescent satellite cells. When satellite cells undergo activation, Myf5 transcript is released for rapid translation, Myf5 protein accumulation occurs, and myogenic differentiation can progress^38^. In our system, it is possible that de novo Myf5 transcription is very low, with most gene regulation occurring post-transcriptionally. Additionally, Myf5 is not the only MRF reported undergoing post-transcriptional regulation. MyoD is a target of multiple RNA binding proteins, including HuR (an mRNA stabilizing factor) and Tristetraprolin (TPP, an mRNA decay factor), allowing for precise control of MyoD transcript levels, and thus the timing of MyoD protein induction and satellite cell activation. In one example, TTP is inactivated via p38/MAPK signaling to permit translation and rapid accumulation of MyoD protein^39^. We postulate that in the context of iPSC-based myogenic differentiation, post-transcriptional (and likely post-translational) gene regulation plays major role in MRF transcript and protein expression. Future studies, however, are needed to confirm this hypothesis and determine the full extent of MRF gene regulation in differentiating iMPCs.

Despite subtle differences between the mRNA and protein studies, both data sets clearly point to substantial deficiencies in myogenic differentiation. Our immunofluorescence data show altered expression of MRFs (Fig. 4), which ultimately culminate in an inability of GNE^1^-derived stage 3 cells to express myosin heavy chain (Figs. 3d and 4j, k). Additionally, MyoG appears to exhibit cytoplasmic localization in some GNE cells, a phenomenon rarely seen in control samples (Fig. 4j and Supplementary Fig. 3E). These studies are backed by scRNAseq analysis corroborating myogenesis defects at a global transcriptional level and qRT-PCR assessment of specific MRF transcript levels (Figs. 5–7, Supplementary Figs. 4–6, and Supplementary Fig. 8). Notably, controls of either male or female origin presented similarly with respect to MRF expression and the ability to differentiate into myotubes (Figs. 3, 4 and Supplementary Figs. 2, 3). Further, our data indicate that observed myogenesis defects in GNEM iMPCs are likely not a general feature of inherited muscle diseases as iMPCs from a nemaline myopathy patient do not demonstrate similar myogenic defects (Supplementary Fig. 2).

The discovery of myogenesis and autophagy defects in GNEM-derived cells has significant clinical implications. Accumulating evidence in other muscle pathology contexts suggests that targeting muscle regeneration and/or autophagy can have a therapeutic benefit. For example, multiple studies investigating age-associated sarcopenia report defects in muscle regeneration^44,45^. Defective p38α/β signaling in muscle satellite cells appears to be a major driver of this dysfunction, and inhibition of the p38/MAPK signaling axis is sufficient to boost the self-renewal of aged stem cells and improve muscle regeneration^44,45^. It has also been shown that activating autophagy in a mouse model of collagen VI muscular dystrophy (Col6a1^−/−^) can improve muscle phenotypes^46^. Under normal conditions, Col6a1^-/-^ muscle was found to have decreased conversion of LC3B-I to LC3B-II compared to control mice, suggesting alterations in basal autophagy levels^46^. Further, when control and Col6a1^-/-^ mice were starved for 24 h, there was an impaired ability to convert LC3B-I to LC3B-II^46^. This group subsequently used prolonged starvation or a low-protein diet to activate autophagy in Col6a1^−/−^ muscle, whereby they found improvement in the number of myofibers containing altered mitochondria and decreased the number of apoptotic nuclei^46^. Further, in their long-term autophagy induction model, tetanic force was increased in Col6a1^−/−^ gastrocnemius muscle^46^. Regeneration-targeting interventions are also beneficial in the context of Duchenne muscular dystrophy (DMD). In a DMD mouse model, satellite cell transplantation can alleviate several DMD pathological hallmarks, including reductions in maximal isometric tetanic force, specific force, myofiber size/number, and dystrophin expression^47^. In a second DMD study, *mdx* mice were treated with an AMPK activator, AICAR (5-aminoimidazole-4-carboxamide-1-β-d-ribofuranoside), causing activation of the autophagy pathway in the diaphragm^48^. *Mdx* mice treated with AICAR were found to have a 21% increase in maximal tectonic force production compared to untreated *mdx* mice^48^. Together, these studies underscore the potential of targeting muscle regeneration and autophagy to improve muscle histopathology and possibly, muscle physiology. Our data also suggest that this approach could and should be further explored in the context of GNEM, especially as differences in sialic acid levels are not clearly/reproducibly evident in GNEM iMPCs, mouse models, or patient tissues. In further support of the role of abnormal autophagy in GNEM, Malicdan and colleagues demonstrate that the rimmed vacuoles found in muscle from the GNEM mouse model containing a *GNE* null allele with transgenic expression of a human GNE D176V (will be referred to as GNE D176V), are different stages of new, degradative, and/or potentially ruptured autophagic vacuoles^49^. The authors also observed that in muscle fibers of normal appearance, amyloid deposits can be present, subsequently lending to a hypothesis in which protein aggregation/amyloid deposits may trigger autophagy^49^. While we believe this is a mechanistic possibility, our data do not support the hypothesis that hyposialylation serves as an upstream trigger leading to the activation of autophagy^49^ as we did not observe differences in overall sialic acid levels (Supplementary Fig. 7) in our model. That said, we acknowledge that further investigation is warranted to clarify the mechanism(s) leading to the apparent autophagy dysfunction in GNEM, and its possible relation to observed regenerative defects described herein.

## Methods

### Human muscle samples

Muscle biopsies were obtained from patients for diagnostic purposes, and conventional histochemical studies were performed on 10 μm thick fresh-frozen tissue muscle sections^50^. Sections were stained for hematoxylin-eosin (H&E), Congo red (CR), acid phosphatase (AcP), and p62/sequestosome (Abcam, dilution 1:200), which was visualized with secondary antibodies using immunoperoxidase. The use of residual diagnostic muscle biopsy tissues was approved by the Mayo Clinic Institutional Review Board (IRB 13-007054 (MM)); skin biopsies were collected and de-identified as described and approved by the Mayo Clinic IRB (IRB 15-006983 (MM) & 13-007298 (DW)).

### Patient biopsies and fibroblast isolation

The biopsy site, located on the inner-upper arm, was cleaned with alcohol wipes, and a local anesthetic was applied. The site was scrubbed with an antimicrobial solution (chloroprep), and a 4 mm punch biopsy was obtained and stored in PBS or DMEM with 1% pen/strep. The tissue was washed using DPBS and then minced with 100 µL 0.25% trypsin. The tissue was collected in a conical tube and centrifuged for 5 min at 800 × *g*, supernatant aspirated, and suspended in 6 mL fibroblast media. Two T-25 flasks were coated with 0.1% gelatin, and the resuspended sample was split between the two flasks and incubated for 14 days at 37 °C. After the initial 14 days, the media was changed 2 times (2×) per week with 5 mL fibroblast medium. After 7–10 days, the media was aspirated, 2 mL of TrypLE was added, cells incubated at 37 °C for 5–10 min, and the reaction quenched with 5 mL fibroblast media. Cells were collected into conical tubes, centrifuged for 5 min at 800 × *g*, supernatant aspirated, and cells resuspended in 10 mL fibroblast media. 5 mL cells were plated into T-75 flasks containing 15 mL fibroblast medium, incubated at 37 °C with a media change 2× per week. Once cells became 80–90% confluent, they were harvested and plated (as stated above) in T-150 flasks containing 20 mL fibroblast media and fed 2× per week with new media. Reprogramming was performed once cells reached 80–90% confluency.

### iPSC reprogramming

Fibroblasts were reprogrammed using Sendai virus-Cytotune 2.0 as described by the manufacturer following the Reprogram fibroblasts Feeder-Free instructions^51^. Fibroblasts were transduced using the CytoTune 2.0 Sendai reprogramming vectors and incubated at 37 °C overnight. The next day, the media was changed to new fibroblast media, and then for 6 days, the fibroblast medium was changed every other day. Vitronectin was used to coat plates, and the fibroblasts were harvested and plated at 2 × 10^4^–1 × 10^5^ and incubated overnight. Media was then changed to complete Essential8 Medium (Thermo) and changed every day for the next ~20 days. Cultures were monitored for iPSC colonies which were picked and cultured using new vitronectin-coated dishes for expansion.

### iPSC culture and quality control

iPSCs were maintained on Geltrex (Thermo Fisher)-coated plates in mTeSR1 (STEMCELL Technologies) or mTeSR Plus (STEMCELL Technologies). Passaging occurred every 3–4 days using ReLeSR (STEMCELL Technologies) and DPBS 1× (Gibco) for washing. Germ layer differentiation^52^ performed by plating iPSCs at a density of 200,000 cells/cm^2^ for ectoderm and endoderm differentiation and 100,000 cells/cm^2^ for mesoderm. Ectoderm Medium (STEMCELL Technologies) with 10 µM Y-27632 (STEMCELL Technologies) was used as a plating medium for cells undergoing ectoderm differentiation. For cells undergoing mesoderm or endoderm differentiation, the plating media used included mTESR1 with 10 µM Y-27632. After 24 h, plating mediums were changed to ectoderm, mesoderm, or endoderm mediums (STEMCELL Technologies) respectively. Media change occurred every day for 5 days for mesoderm and endoderm and 7 days for ectoderm.

### iPSC/iMPC myogenic differentiation

Skeletal muscle differentiation^53^ occurred in a three-stage process: (1) iPSCs were differentiated to myogenic precursors by dissociating iPSCs with Accutase (Sigma) for 5 min, resuspended in Skeletal Muscle Induction Media (Stage 1 media) (Genea Biocells), centrifuged at ~300 × *g* for 2 min, decanted, and suspended in Stage 1 media. Cells were counted and plated in BioCoat collagen I 24-well plates (Corning) or collagen I (Gibco) coated plates at 7500 cells/cm^2^. Stage 1 media was changed every 2 days until cells reached confluence, (2) myogenic precursor cells were further differentiated to myoblasts by dissociation of myogenic precursor cells using 0.05% Trypsin-EDTA for 5 min, trypsin was neutralized using 10% FBS (Gibco) in DMEM (Gibco), cells centrifuged for 4 min at ~300 × *g*, and resuspended in Skeletal Myoblast Medium (Stage 2 media) (Genea Biocells). Cells were counted and plated in 24-well Collagen I coated plates at 10,000 cells/cm^2^. Stage 2 media was changed every 2 days until cells reached confluency. (3) Myoblasts were differentiated to myotubes by changing Stage 2 media to Skeletal Myoblast Medium (Stage 3 media) (Genea Biocells). Stage 3 media was changed every 2 days until the formation of myotubes. SB203580 drug treatment: GNE patient cells were differentiated as stated above. At the first change of Stage 2 media, either vehicle, 0.1, 0.5, 1, or 2.5 μM SB203580 was added to replicate wells. Drug treatments were continued for each subsequent media change through Stage 3, until the formation of myotubes and subsequent fixation.

### Sanger sequencing/mutation confirmation

iPSC samples were used for DNA isolation using the DNeasy Blood and Tissue Kit (Qiagen). PCR was performed using PCR SuperMix (Thermo) following the manufacturer’s instructions. All PCR products were enzymatically cleaned using ExoSap-IT (Thermo). Sequencing primers were added, and samples were sent to GeneWiz for Sanger sequencing. 4Peaks software was used for trace visualization and the presence of mutation verification.

Primers were purchased from Integrated DNA Technology (IDT) and the sequences used are as follows (Forward/Reverse/Sequencing Primer):

GNEc.479 G > A: 5’-CCAGGCTACACACAATTGTGAGGGG-3’/5’-GAAGTTTGTCATAGGAAGGGCAGCC-3’/5’-GCTGCCAGATGTCCTTAATCGCCTG-3’

GNEc.1835G > T: 5’-GATGCCTAGTGGGCTTCAGCTGTC-3’/5’-CCACCACCACCCCTGGGGAGG-3’/5’-CCCTCGCTGTAGGAATCGGTGGTG G-3’

GNEc.2179 G > A and c.2218 G > A: 5’-GCCTCCCACTGCATGCCGTGG-3’/5’-GTGGGGAAAGGTGACTCTGGAAGAGG-3’/5’-CCCTCCCTTGTGATCCTCTCCGG-3’.

### Immunofluorescence

Cultured cells from the indicated time points were washed with PBS and fixed in 24-well plates using 4% Paraformaldehyde Solution (Santa Cruz) for 20 min at room temperature, washed 2× with PBS, and stored in PBS at 4 °C until ready for use. Cells were permeabilized using 0.2% Triton X-100 (Fisher) in 1:10 dilution Blocking Buffer (Dako) for 30 min at room temperature. Cells were washed for 5 min using 1× Wash Buffer and then blocked by adding 8 drops of Protein Block Serum-Free (Agilent). Primary antibodies were diluted in diluent (Dako) and incubated at room temperature for 1 h, washed 2× with Wash Buffer for 5 min, and incubated with secondary antibodies in diluent for 30 min at room temperature. Cells were washed 2× with Wash Buffer for 5 min, incubated with Nunc Blue (Invitrogen −2 drops/mL in PBS) for 5 min, washed with PBS, and stored in PBS at 4 °C for up to 2 weeks for imaging. See antibody details in Supplemental Table 2. Images were taken with BioTek Cytation5 imaging reader using Gen5 (v3.06) software. SB203580 treated cell immunofluorescence: Vehicle or SB203580 drug treated GNE myotubes were washed with PBS and fixed in 24-well plates using 4% Paraformaldehyde Solution (Thermo Fisher) for 15 min at room temperature, washed 2× with PBS, and then stored in PBS at 4 °C until ready for staining. Cells were permeabilized using 0.5% Triton X-100 (Sigma) in PBS for 5 min at room temperature. Cells were washed 2× for 5 min using an immunofluorescence buffer (IFB: 3% Albumin Bovine Serum (Goldbio), 0.2% Triton X-100, 0.2% Tween-20 (Sigma), in PBS) and then blocked with IFB for 60 min at room temperature. Primary antibodies were diluted in IFB and wells incubated at room temperature for 90 min, washed 2× with IFB for 5 min, and incubated with secondary antibodies diluted in IFB for 45 min at room temperature. Cells were washed 2× with IFB for 5 min, incubated with DAPI diluted in IFB or Nuclear ID for 10 min, washed with PBS, and stored in PBS at 4 °C for up to 2 weeks for imaging. See antibody details in Supplemental Table 4. Images were taken with the Hamamatsu ORCA-Flash 4.0 LT CMOS camera using Nikon NIS-Elements.

### Image analysis

Cell Profiler (version 3.1.9)^54^ was used to quantify nuclei-positive cells by setting the diameter and upper/lower thresholds in control images and applying the same settings to the GNE images in samples run under similar conditions, except for stage 3 MyoG nuclei-positive cells (Fig. 4i) which were manually quantified. LAMP1 nuclear engulfment (Fig. 3c) and stage 3 quantification of myotube numbers (Fig. 4k) was quantified manually. Each n, represented as a single point on the graph in Fig. 3c and 4, is the average value from the quantification of 4–5 images per well across all available clones per line. Incucyte ZOOM 2016B was used for quantification of a number of green and phase object counts per image in Fig. 8c, e, where *n* = 1 is the average of 16 or 36 images per well.

### qRT-PCR

RNA was isolated from cells using TRIzol (Life Technologies). Samples were mixed with chloroform, centrifuged at ~16,000 × *g* for 5 min, and supernatant taken through the Qiagen RNeasy Mini Kit following the manufacturing handbook. Briefly, the supernatant was mixed 1:1 with 70% ethanol, placed in the RNeasy spin column, and centrifuged for 15 s at ~9500 × *g*. The column was washed with Buffer RW1, and twice with Buffer RPE with a 15 s centrifuge in between each step at ~9500 × *g*. RNA was eluted with Nuclease-Free Water (Invitrogen), and RNA concentration was determined using a NanoDrop Spectrophotometer (Thermo ND-1000). Two micrograms RNA per reaction was transcribed using the High Capacity cDNA Reverse Transcription Kit (Applied Biosystems) according to the manufacturer’s protocol. qRT-PCR was performed using SsoAdvanced Universal SYBR Green Supermix (Bio-Rad) in accordance with manufacturer protocol using the ViiA7 Quantitative PCR System (Applied Biosystems). All samples were run in either a technical triplicate or quadruplet. Primers were identified via the online Harvard primer bank^55^ (https://pga.mgh.harvard.edu/primerbank/) and purchased from IDT as follows (Forward/Reverse):

*Β-Actin:* 5’-CATGTACGTTGCTATCCAGGC-3’/5’-CTCCTTAATGTCACGCACGAT-3’

*Pax3:* 5’-CCGGGGCAGAATTACCCAC-3’/5’-GCCGTTGATAAATACTCCTCCG-3’

*Myf5:* 5’-AAGGCTCCTGTATCCCCTCAC-3’/5’-TGACCTTCTTCAGGCGTCTAC-3’

*MyoD:* 5’-CCACTCCGGGACATAGACTTG-3’/5’-AAAAGCGCAGGTCTGGTGAG-3’

*MyoG:* 5’-GAGACATCCCCCTATTTCTACCA-3’/5’-GCTCAGTCCGCTCATAGCC-3’

### Single-cell mRNA sequencing

Three wells, from a 24-well plate (Corning) of cells, were scraped and combined from the indicated time points and suspended in DPBS. Cells were then assessed for viability and count using Vi CELL XR (Beckman Coulter) analysis version 2.42. Samples with the highest cell count and viability were selected for further single-cell RNA sequencing processing. 10× Genomics single-cell reagent 3’ v2 was used to prepare the cDNA libraries and then were sequenced on the Illumina HiSeq 4000, with paired-end 100 bp reads. 10× Genomics Cell Ranger Single Cell Software Suite (v2.2.0) was used to demultiplex raw base call (BCL) files generated from the sequencer into FASTQ files (Cellranger mkfastq command) and to perform alignment to the hg38 genome, filtering, barcode counting and UMI counting (Cellranger count command). The gene expression matrices files were used for the subsequent analyses. Seurat version 4.0.1^56^ (https://github.com/satijalab/seurat/releases/tag/v4.0.1) was used in RStudio (http://www.rstudio.com/. Version 1.3.959. Release Name: Middlemist Red) using R Version 4.0.2 (https://cran.r-project.org/bin/windows/base/old/4.0.2/. Release name: Taking off Again.) to analyze data and filter out cells that were poor quality. Coding was performed predominately following the Satija lab Seurat tutorial (https://satijalab.org/seurat/v3.1/pbmc3k_tutorial.html). Each sample was assessed individually, and threshold values were assigned for low unique molecular identifiers (threshold 1800–10,000), low gene detection (threshold 900–3000), and high mitochondrial transcript fraction (threshold 10–25%). All data was (i) normalized using NormalizeData, (ii) scaled using ScaleData, (iii) run with principal component analysis (PCA) using the RunPCA command, and (iv) UMAP using RunUMAP.

Pseudotime trajectory was performed using Seurat version 4.0.2^56^ (https://github.com/satijalab/seurat/releases/tag/v4.0.2) in RStudio (http://www.rstudio.com/. Version 1.4.1106.4. Release name: Tiger Daylily), R Version 4.0.2 (https://cran.rproject.org/bin/windows/base/old/4.0.2/. Release name: Bunny-Wunnies Freak Out), with monocle version 2.18.0^57–59^ essentially as described (https://cole-trapnell-lab.github.io/).

Hierarchical clustering was performed by TIBCO Spotfire Analyst 7.11.2.4 using average log2 gene expression for each time point for the top 100 differentially expressed genes as found by the R command FindAllMarkers, with expression in a minimum of 25% cells and greater than a log fold-change of 0.25.

### Western blotting

Protein was isolated from cells washed with PBS 2× using the RIPA Lysis Buffer System (ChemCruz) and concentration was determined using the Bradford Assay (Bio-Rad), both according to manufacturer instructions. Western blotting was performed by loading 15 μg protein per sample into 4–20% Mini-PROTEAN TGX gels or 10% Mini-PROTEAN TGX gels for SNA staining (Bio-Rad). Proteins were transferred to a PVDF membrane (Bio-Rad), and Ponceau S solution (Sigma) was used to quantify total protein. Five percent blotting-grade blocker (Bio-Rad) or 5% BSA (GoldBio) was used for blocking for 1 h at room temperature, and then membranes were probed with primary antibodies (listed in Supplementary Table 5) overnight at 4 °C with agitation. Secondary antibodies (Supplementary Table 5) were incubated for ~1 h at room temperature, and pierce ECL western blotting substrate was used to visualize bands. All bands were quantified using volume intensity from Image Lab 6.1 software. Representative examples of blots were included in the main figures. Uncropped/unprocessed blots from Figs. 7, 8, and Supplementary Fig. 7 can be found in Supplementary Figs. 11 and 12.

### Statistical analyses

GraphPad Prism 8 (version 8.4.2) or 9 (versions 9.0.0-9.3.0) was used for all statistical analyses. Student’s *t*-test were unpaired and significance indicated with *p < 0.05, **p < 0.01, ***p < 0.001. For correlation analyses, Pearson’s correlation coefficient was used. All numerical data are reported as mean ± standard error of the mean (SEM).

### Reporting summary

Further information on research design is available in the Nature Research Reporting Summary linked to this article.

## Supplementary information

Supplementary Material

Reporting Summary

## Data Availability

Data from this study are available in the Sequence Read Archive (Accession Number PRJNA739168).
