# Supplementary material for: Myogenesis defects in a patient-derived iPSC model of hereditary GNE myopathy
Source: NPJ Regen Med. 2022 Sep 9;7:48. doi: 10.1038/s41536-022-00238-3 (PMC9463157; doi:10.1038/s41536-022-00238-3)
Supplement: Supplementary file 1 — Supplementary Material [file 41536_2022_238_MOESM1_ESM.pdf]

Supplementary Figure 1

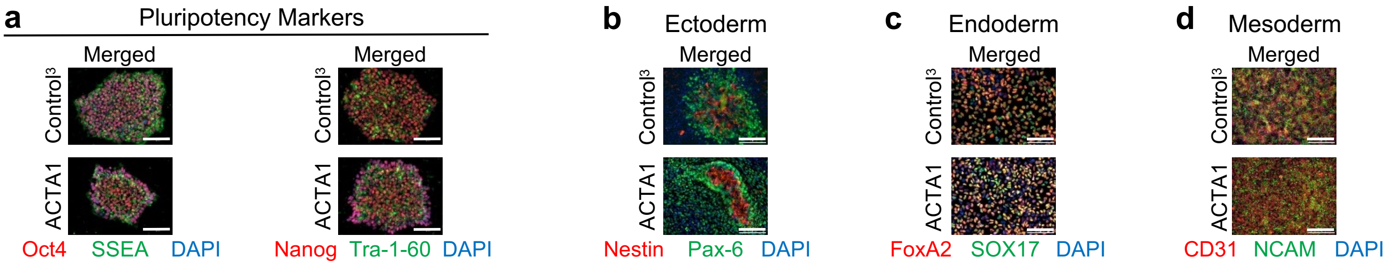

Supplementary Figure 1. ***Pluripotency markers and germ layer differentiation of male control and Nemaline myopathy patient-derived iPSCs.*** (A) Male control (Control[3]) and Nemaline myopathy (ACTA1) patient-derived iPSCs expressed pluripotency markers Oct4, SSEA, Nanog, and Tra-1-60. (B-C) IF images highlighting germline differentiation capacity. Differentiated Control[3] and ACTA1 iPSCs were capable of expressing standard ectoderm (Nestin and Pax-6) (B), endoderm (FoxA2 and SOX17) (C), and mesoderm (CD31 and NCAM) markers (D). All images are representative. Images are 20X, scale bars: 100 μm.

Supplementary Figure 2

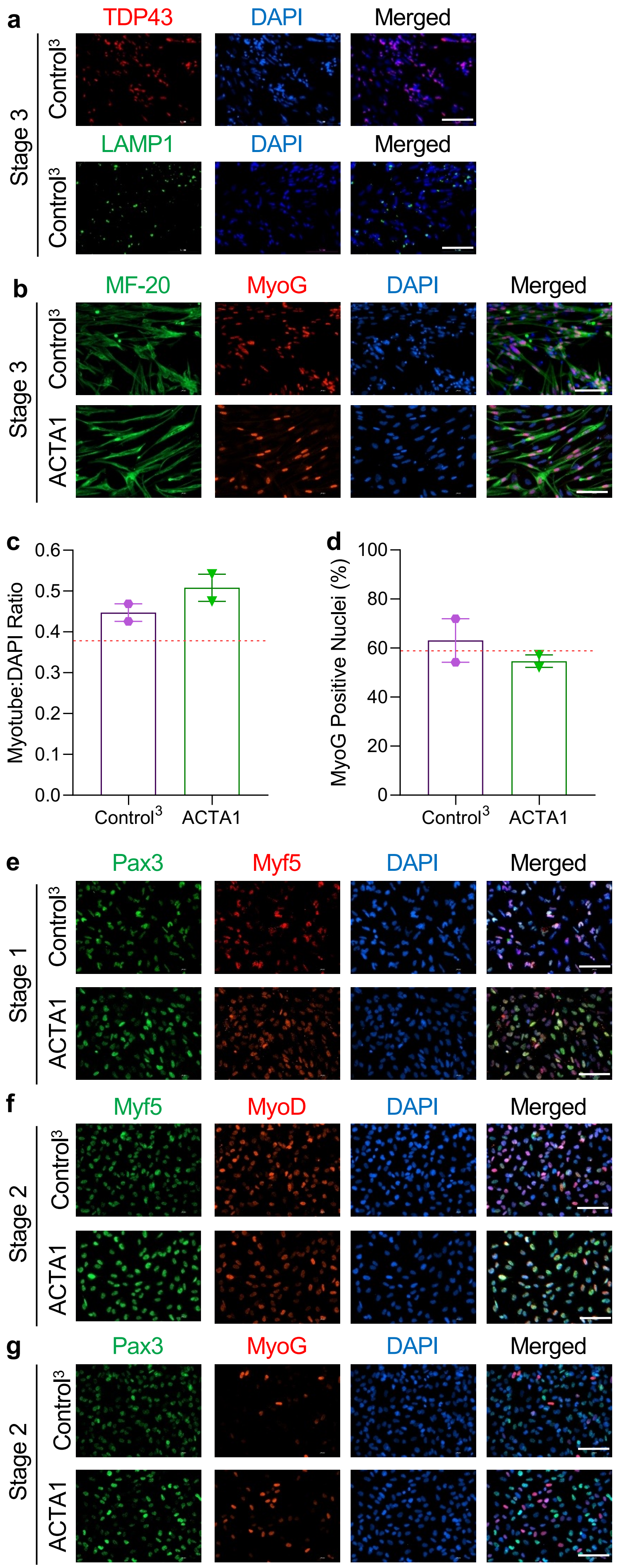

Supplementary Figure 2. ***GNE characteristics, myotube differentiation, and MRF expression in a male origin control iPSC line and a Nemaline myopathy patient-derived iPSC line.*** (A) IF images depicting GNE markers in differentiated myotubes of a male origin iPSC line (Control[3]); TDP-43 (red) and DAPI (blue, top) or LAMP1 (green) and DAPI (blue, bottom). (B-G) Control[3] (top) and Nemaline myopathy (ACTA1, bottom) patient-derived iMPCs (stage 1 = E, stage 2 = F-G) were differentiated to myotubes (stage 3, B-D) and stained for MRFs throughout differentiation. (B) Stage 3 IF; MyoG (red), myosin heavy chain (MF-20, green), and DAPI (blue). (C) Quantification of number of myotubes to DAPI ratio per 20X image. (D) Quantification of MyoG percent positive nuclei from Control[3] (purple) and ACTA1 (green). Red dashed line in C and D indicates average levels of myotube:DAPI or MyoG percent positive nuclei, respectively, from Control[1] and Control[2] for comparison. (E) Stage 1 IF; Pax3 (green), Myf5 (red), and DAPI (blue). (F) Stage 2 IF; Myf5 (green), MyoD (red), and DAPI (blue). (G) Stage 2 IF; Pax3 (green), MyoG (red), and DAPI (blue). All images are representative; A-B and E-G were taken at 20X, scale bars: 100 µm. C-D each point in quantification represents n = 1 from the average values of 4-5 images per well, n = 2. All statistical analyses were performed using Student's unpaired t-test where data are presented as mean ± standard error of the mean (SEM). Significance is $*p<0.05$, $**p<0.01$, $***p<0.001$.

Supplementary Figure 3

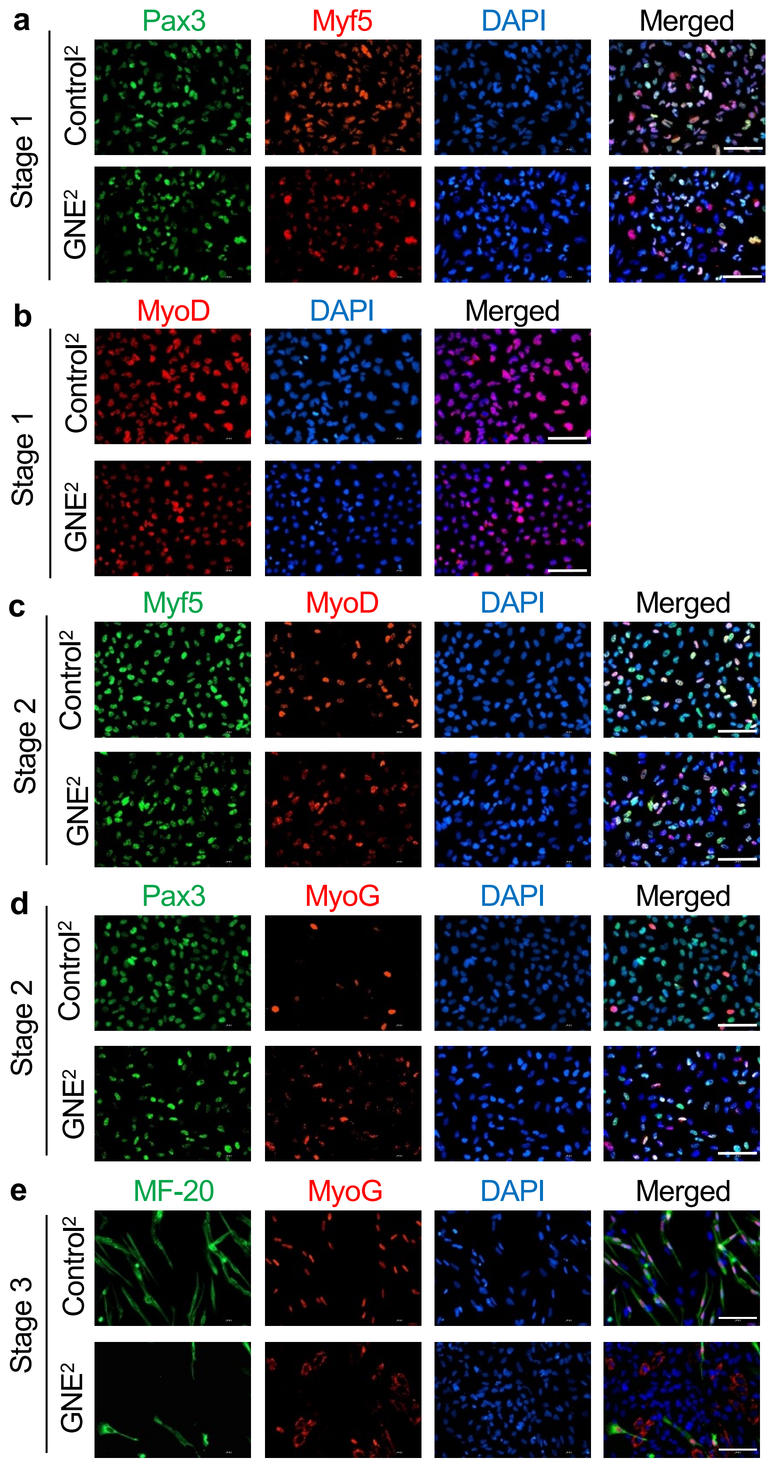

Supplementary Figure 3. ***Representative IF images of Control^{2} and GNE^{2} MRF expression.*** (A-B) Satellite-like cell, or stage 1 iMPCs MRF protein expression in Control^{2} (top) and GNE^{2} (bottom) cells stained with (A) Pax3 (green), Myf5 (red), and DAPI (blue) or (B) MyoD (red) and DAPI (blue). (C-D) Protein expression of myogenic transcription factors of stage 2 iMPCs. Control^{2} (top) and GNE^{2} (bottom) stage 2 cells stained with (C) Myf5 (green), MyoD (red), and DAPI (blue) and (D) Pax3 (green), MyoG (red), and DAPI (blue). (E) Control^{2} (top) and GNE^{2} (bottom) iMPCs were differentiated to myotubes (stage 3) and stained for myogenic markers MyoG (red) and myosin heavy chain (green). Quantification is represented in Figure 4. All images are 20X representative images, scale bar: 100 μm.

Supplementary Figure 4

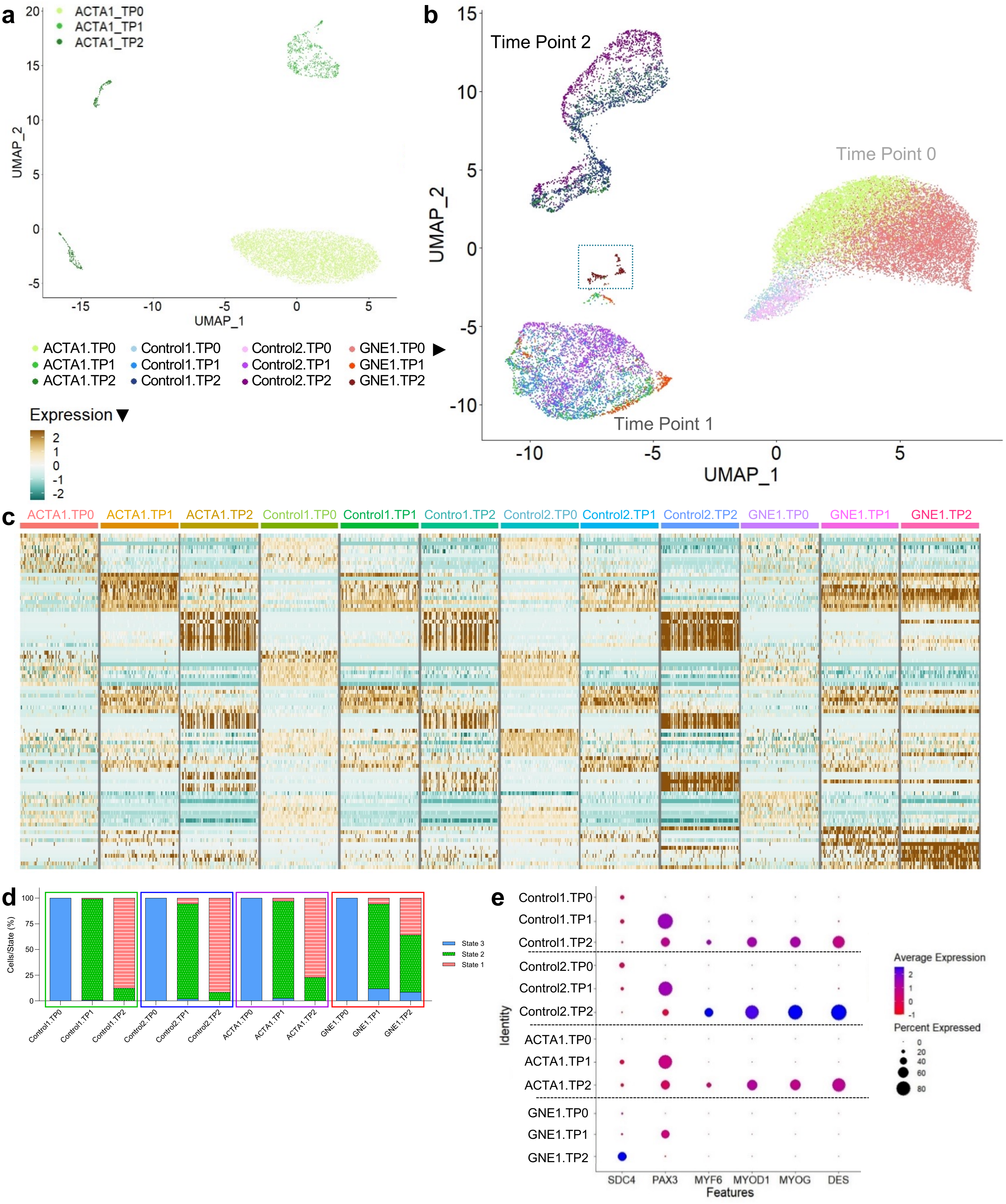

Supplementary Figure 4. ***Global transcriptome comparison between controls, GNEM, and Nemaline myopathy patient-derived iPSCs undergoing myogenic differentiation.*** (A-B) UMAP projection of single cell data from (A) ACTA1 and (B) combined Control^{1} (Control1), Control^{2} (Control2), GNE^{1} (GNE1), and ACTA1 containing Time Points 0, 1, and 2. (C) Heatmap depicting the log scale of the top 100 DEGs, as represented by 200 cells from each group, for ACTA1, Control1, Control2, and GNE1 Time Points 0, 1, and 2. Brown: higher expression and Teal: lower expression. (D) Proportion of cells from each sample that fall within state 1 (red and lined), state 2 (green and dotted), and/or state 3 (blue solid) from pseudotime trajectory analysis across all time points using single cells from ACTA1, Control1, Control2, and GNE1. (E) Dot plots of selected MRFs in ACTA1, Control1, Control2, and GNE1 samples at Time Points 0, 1, and 2. Blue: higher expression, red: lower expression.

Supplementary Figure 5

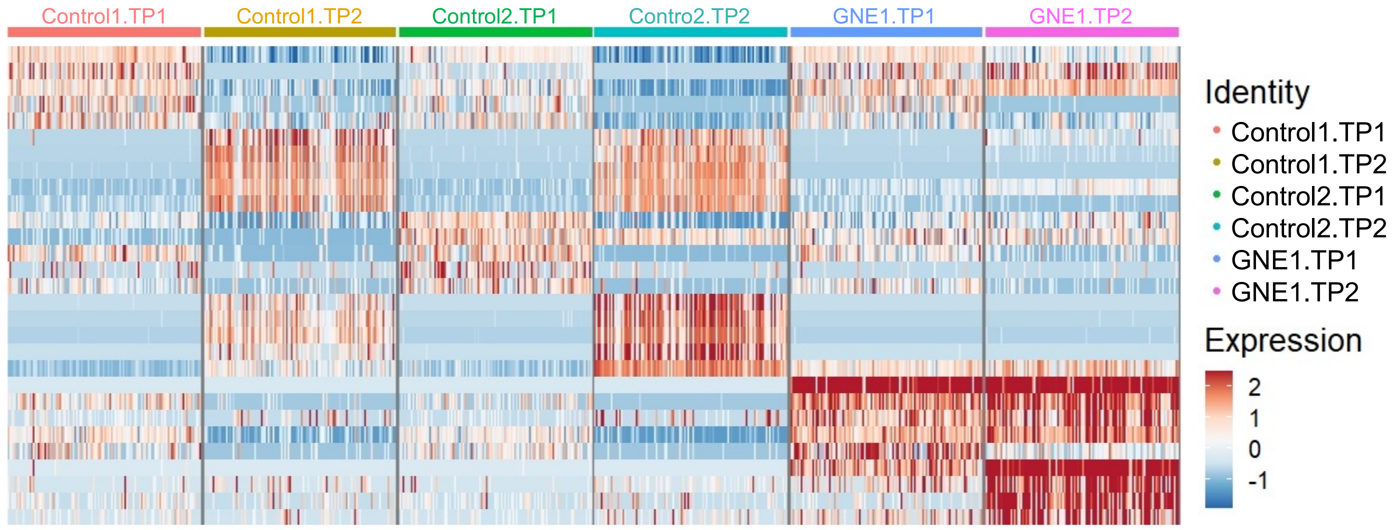

Supplementary Figure 5. ***Heatmap illustrating top 5 DEGs from Control^{1}, Control^{2}, and GNE^{1} at time points 1 and 2.*** Red: high expression and blue: low expression.

Supplementary Figure 6

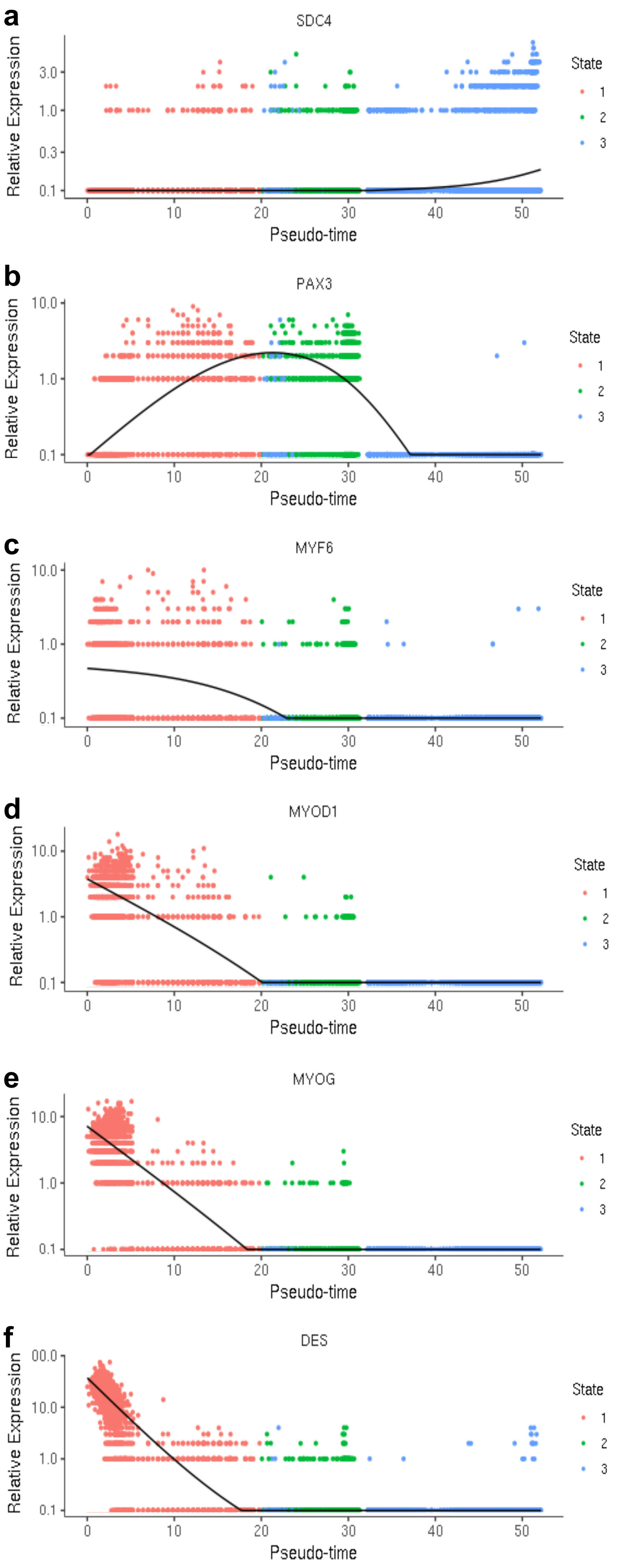

Supplementary Figure 6. ***Confirmation of pseudotime ordering of states by MRF expression patterns.*** (A-F) Specific MRF expression across the painted pseudotime trajectory by state; (A) SDC4, (B) PAX3, (C) MYF6, (D) MYOD1, (E) MYOG, and (F) DES.

Supplementary Figure 7

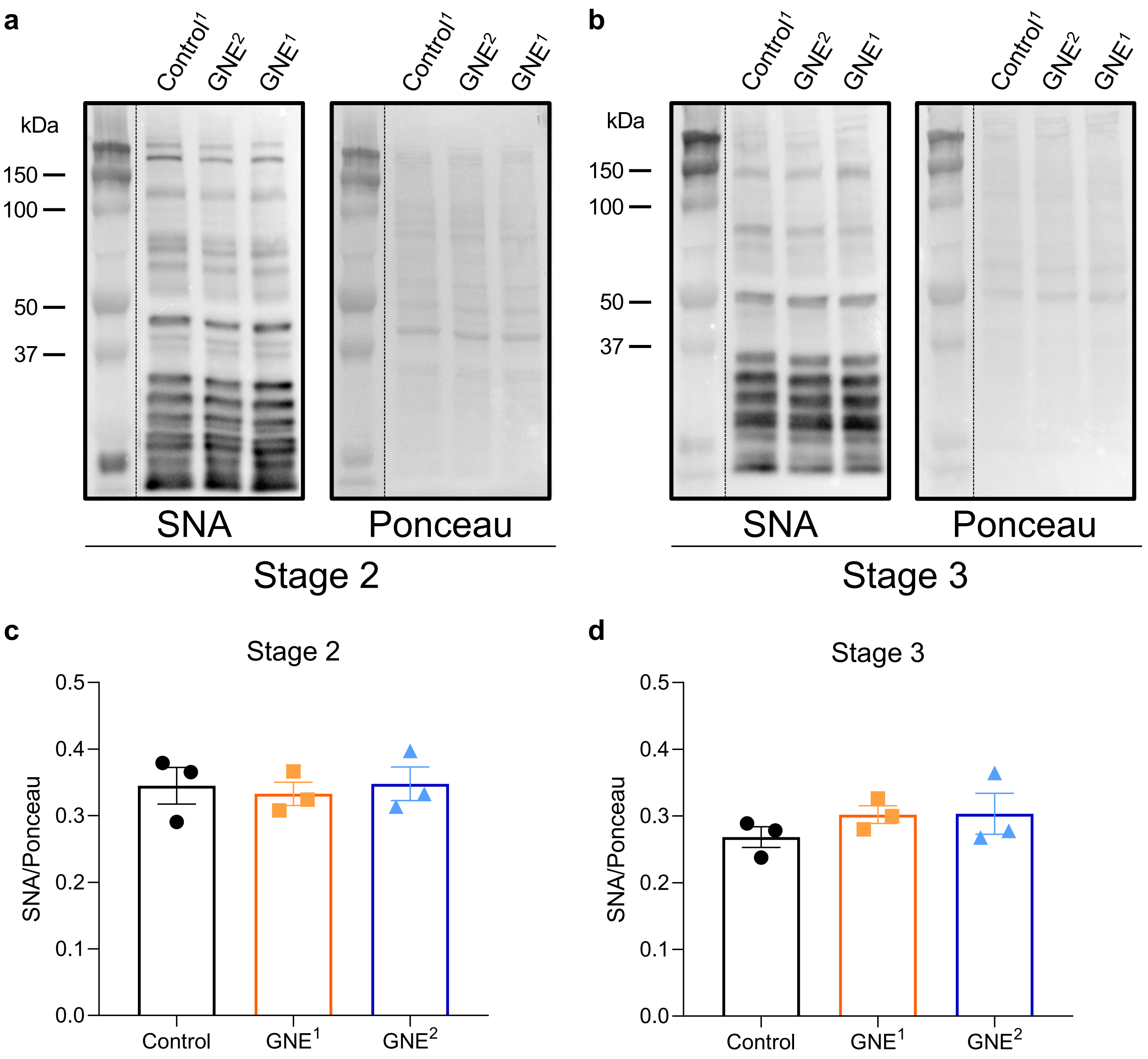

Supplementary Figure 7. ***No apparent alterations in sialic acid levels in stage 2 and 3 GNEM iMPCs.*** (A-D) Measurement of sialic acid levels via SNA lectin western blotting. (A-B) Representative SNA and ponceau staining from Control^{1}, GNE^{1}, and GNE^{2} at stage 2 (A) or stage 3 (B). Ladder was stitched to blots, see original blots in Supplementary Fig. 12. (C-D) Quantification of SNA levels normalized to total protein concentration in Controls (Control^{1} and Control^{2} combined), GNE^{1}, and GNE^{2} at stage 2 (C) and stage 3 (D). C-D statistical analyses performed using Student's unpaired t-test, n = 3, and data are presented as mean ± SEM. Significance is *p<0.05, **p<0.01, ***p<0.001.

Supplementary Figure 8

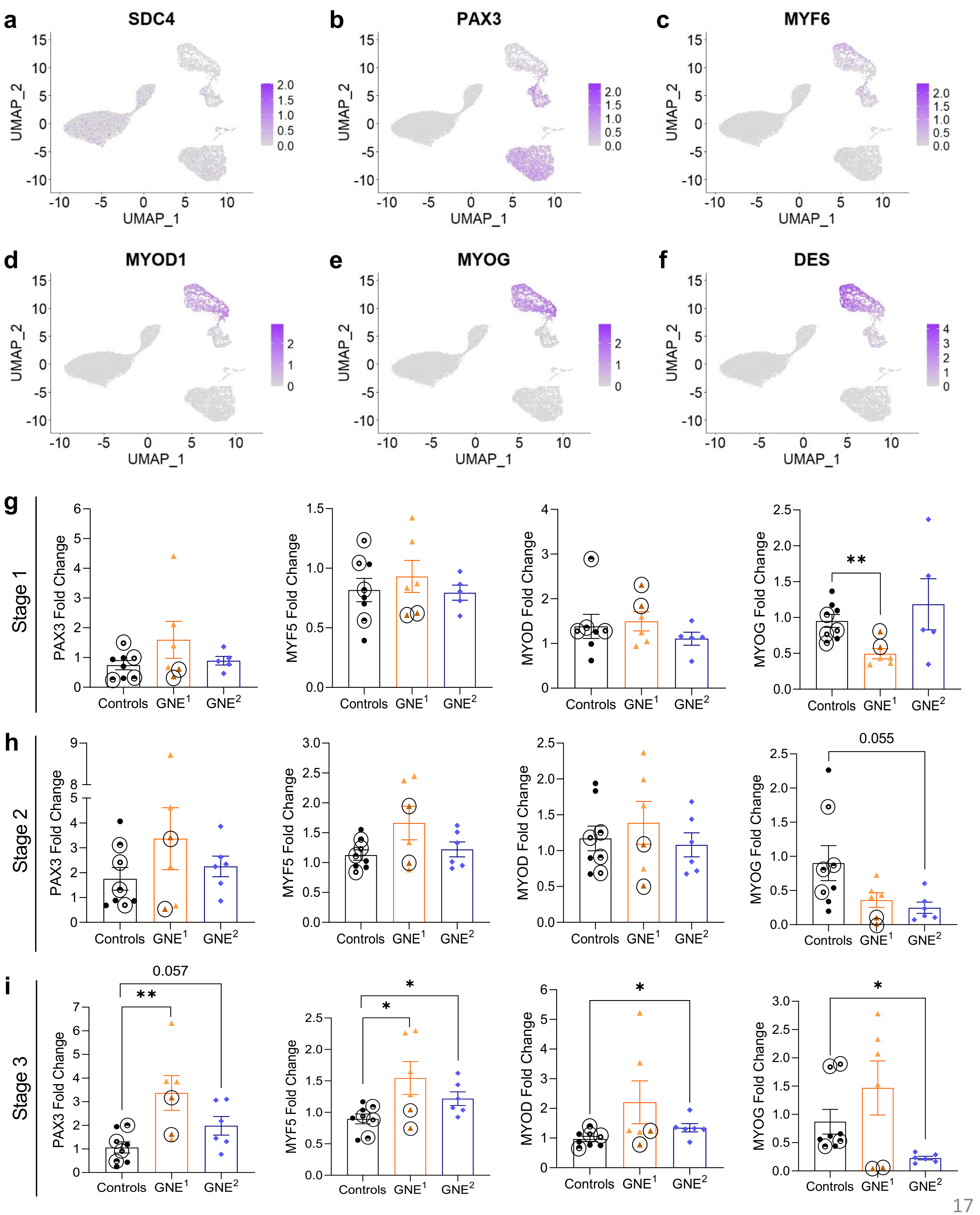

Supplementary Figure 8. ***Altered MRF expression in GNE myopathy iMPCs.*** (A-F) Single-cell expression of selected genes from Figure 5E, depicted via UMAP. (A) SDC4, (B) PAX3, (C) MYF6, (D) MYOD1, (E) MYOG, and (F) DES. Blue: higher expression and gray: lower expression. (G-I) qRT-PCR of PAX3, MYF5, MYOD, and MYOG from Controls (combined Control^{1} and Control^{2}), GNE^{1}, and GNE^{2} clones across stage 1 (G), stage 2 (H), and stage (3). Circled points indicate specific clone used in single-cell RNA sequencing. G-I = Student's unpaired t-test, n = 6-8, and data are presented as mean ± SEM. Significance is *$p<0.05$, **$p<0.01$, ***$p<0.001$.

Supplemental Figure 9

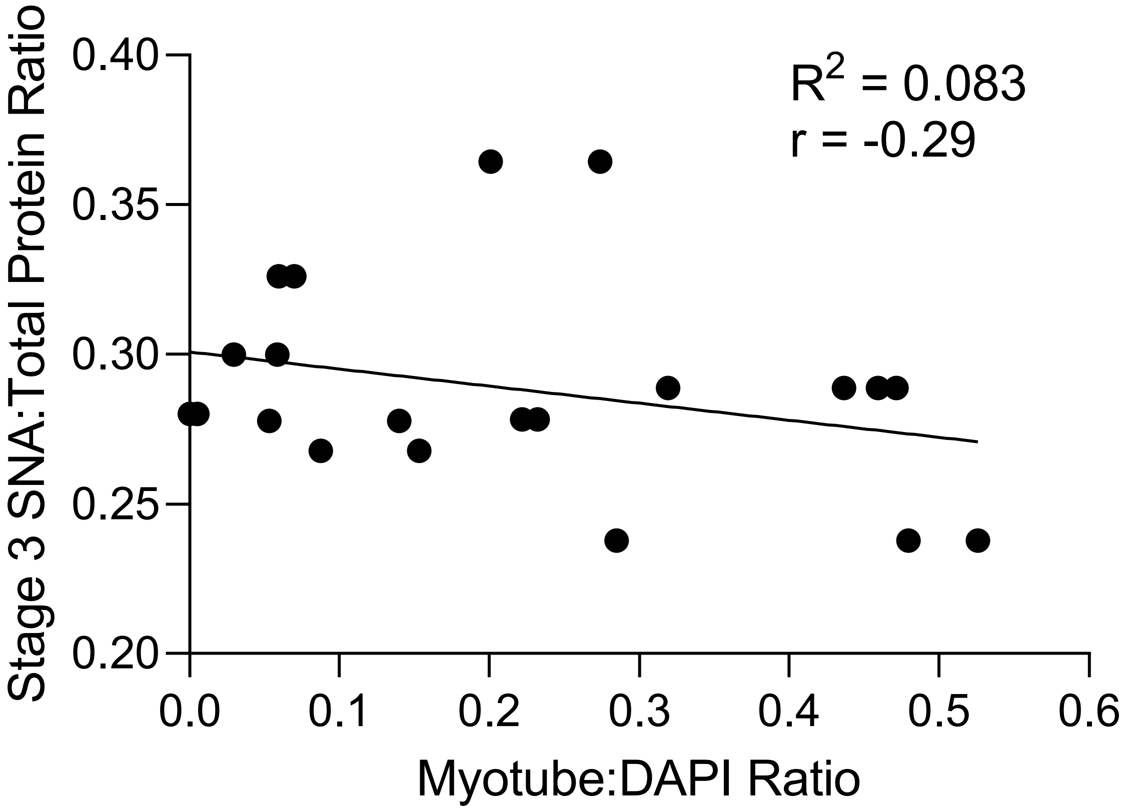

Supplementary Figure 9. ***Absence of correlation between stage 3 SNA levels and ability of iMPCs to form myotubes.*** Correlation analysis of stage 3 SNA levels versus myotube:DAPI ratio (values from S7D and 4K, respectively). Statistical analysis performed was Pearson's correlation analysis. Significance is $*p<0.05$, $**p<0.01$, $***p<0.001$.

Supplementary Figure 10

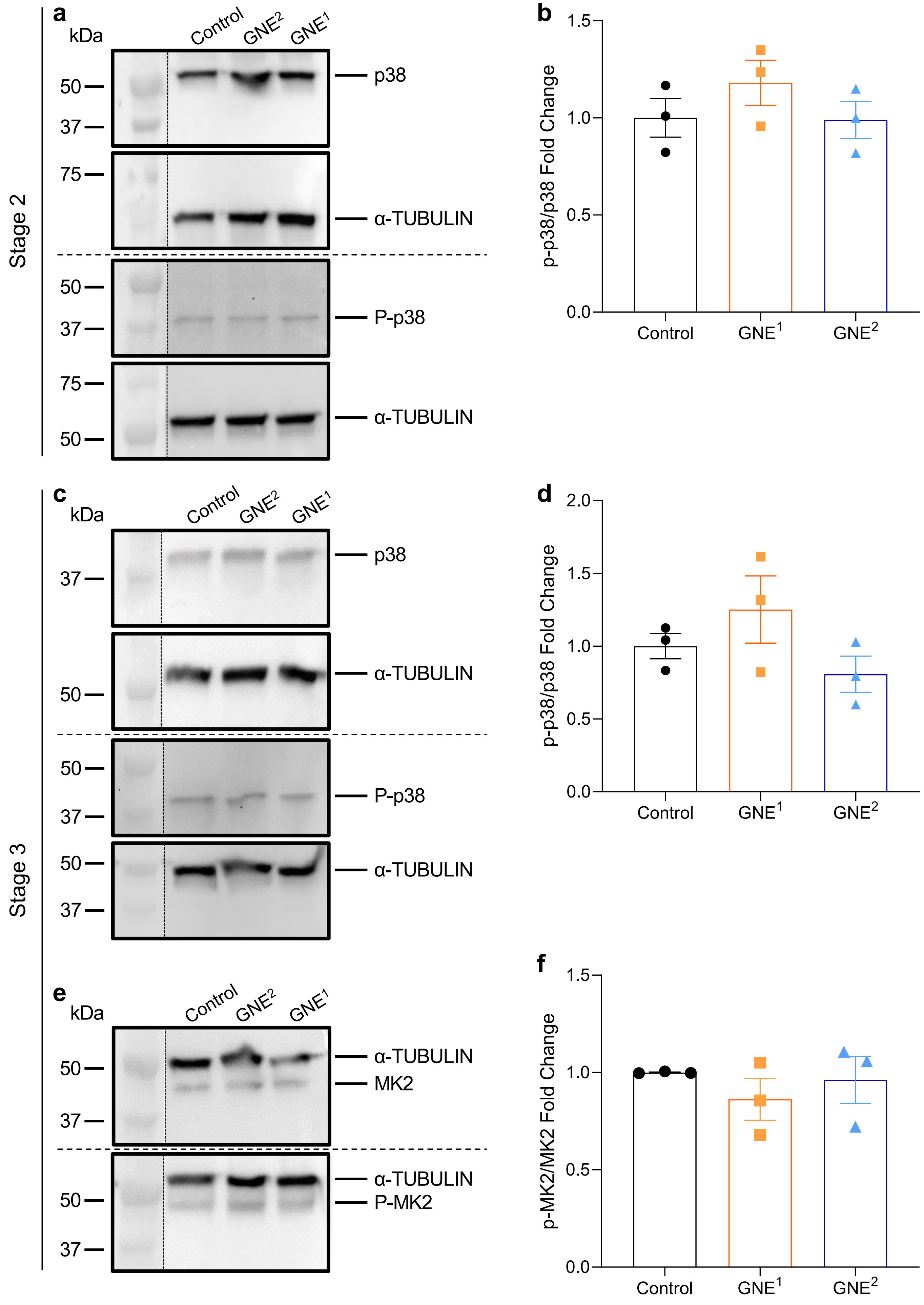

Supplementary Figure 10. ***Activation of p38 signaling is not altered in stage 2 or 3 GNEM iMPCs.*** (A-B) Representative stage 2 western blots of p38 and p-p38 in Control^{1}, GNE^{1}, and GNE^{2} (A) and subsequent quantification of fold change of p-p38/p38 in Controls (combined Control^{1} and Control^{2}), GNE^{1}, and GNE^{2} (B). (C-F) Stage 3 representative blots of either p38 and p-p38 (C) or p38 downstream target MK2 and p-MK2 (E). Respective quantification of p-p38/p38 (D) and p-MK2/MK2 (F) in Controls (combined Control^{1} and Control^{2}), GNE^{1}, and GNE^{2}. Ladder was stitched to blots. B, D, and F = Student's unpaired t-test, n = 3, and data are presented as mean ± SEM. Significance is *$p<0.05$, **$p<0.01$, ***$p<0.001$.

Supplementary Figure 11

a

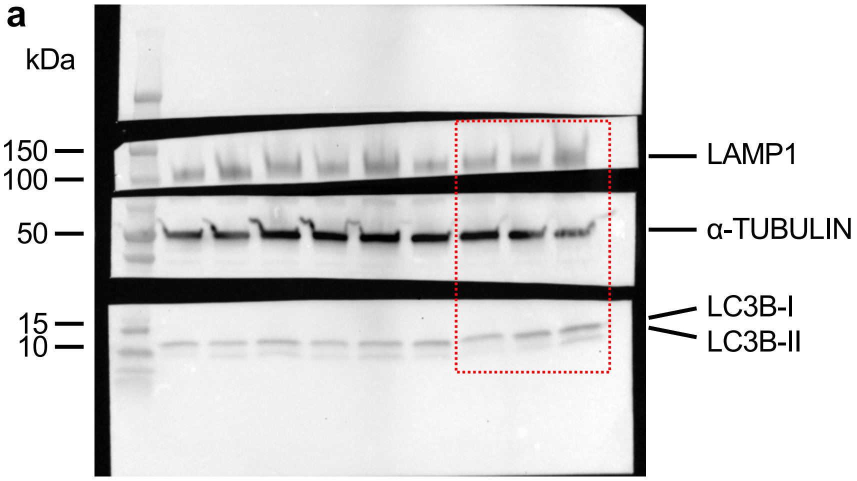

b Stage 3

Lane	Sample
1	Ladder
2	Control$^{2-2}$
3	GNE$^{1-2}$
4	GNE$^{1-3}$
5	GNE$^{2-3}$
6	GNE$^{2-2}$
7	Control$^{1-1}$
8	Control$^{1-2}$
9	GNE$^{2-1}$
10	GNE$^{1-1}$

Lanes: Left #1 → Right #10

c

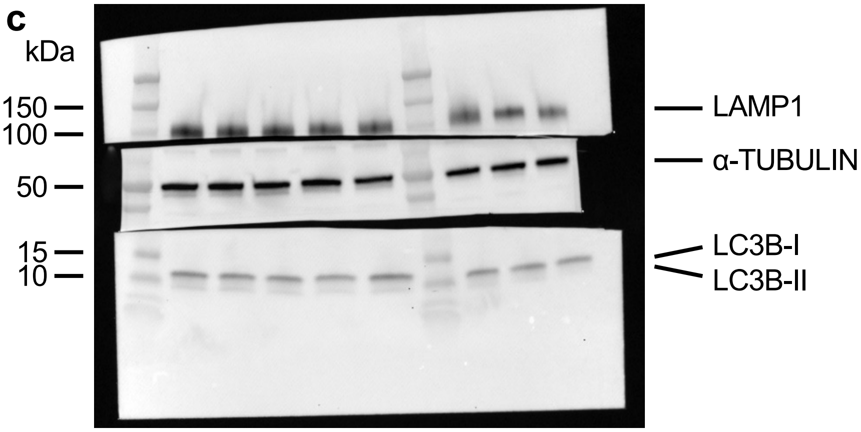

d Stage 3

Lane	Sample
1	Ladder
2	--
3	--
4	--
5	--
6	--
7	Ladder
8	--
9	--
10	Control$^{2-1}$

Lanes: Left #1 → Right #10

e

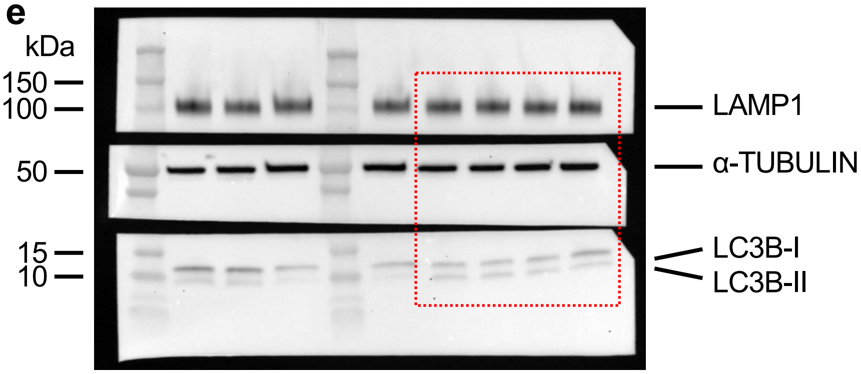

f

Lane	Sample
1	Ladder
2	GNE$^{2-1}$ SB203580 Vehicle
3	GNE$^{2-1}$ SB203580 0.1 μM
4	GNE$^{2-1}$ SB203580 1.0 μM
5	Ladder
6	GNE$^{2-1}$ SB203580 2.5 μM
7	GNE$^{1-1}$ SB203580 Vehicle
8	GNE$^{1-1}$ SB203580 0.1 μM
9	GNE$^{1-1}$ SB203580 1.0 μM
10	GNE$^{1-1}$ SB203580 2.5 μM

Lanes: Left #1 → Right #10

Supplementary Figure 11. ***Unaltered autophagy-related protein blots of control and GNEM iMPCs depicted in main figures 7 and 8.*** (A, C, and E) Blots used for the measurement of LAMP1, α-TUBULIN, LC3B-I, and LC3B-II. (B, D, and F) Designation of samples loaded into each lane (for the blot to the left of the designations (e.g., b is for a)) starting with lane 1 on the far left of each blot and proceeding right. (A and C) Blots used for quantification of stage 3 LC3B-II/I ratio expression from control and GNEM iMPC samples as represented in figure 7G. Red box = location of the cropped representative image found in figure 7F. (E) Blots used for quantification of LAMP1 expression and the LC3B-II/I ratio in GNE^1 and GNE^2 SB203580 treated cells found in figure 8G-H. Red box = location of cropping for representative images of LAMP1 and LC3B-II/I from GNE^1 SB20350 treated cells found in figure 8F.

Supplementary Figure 12

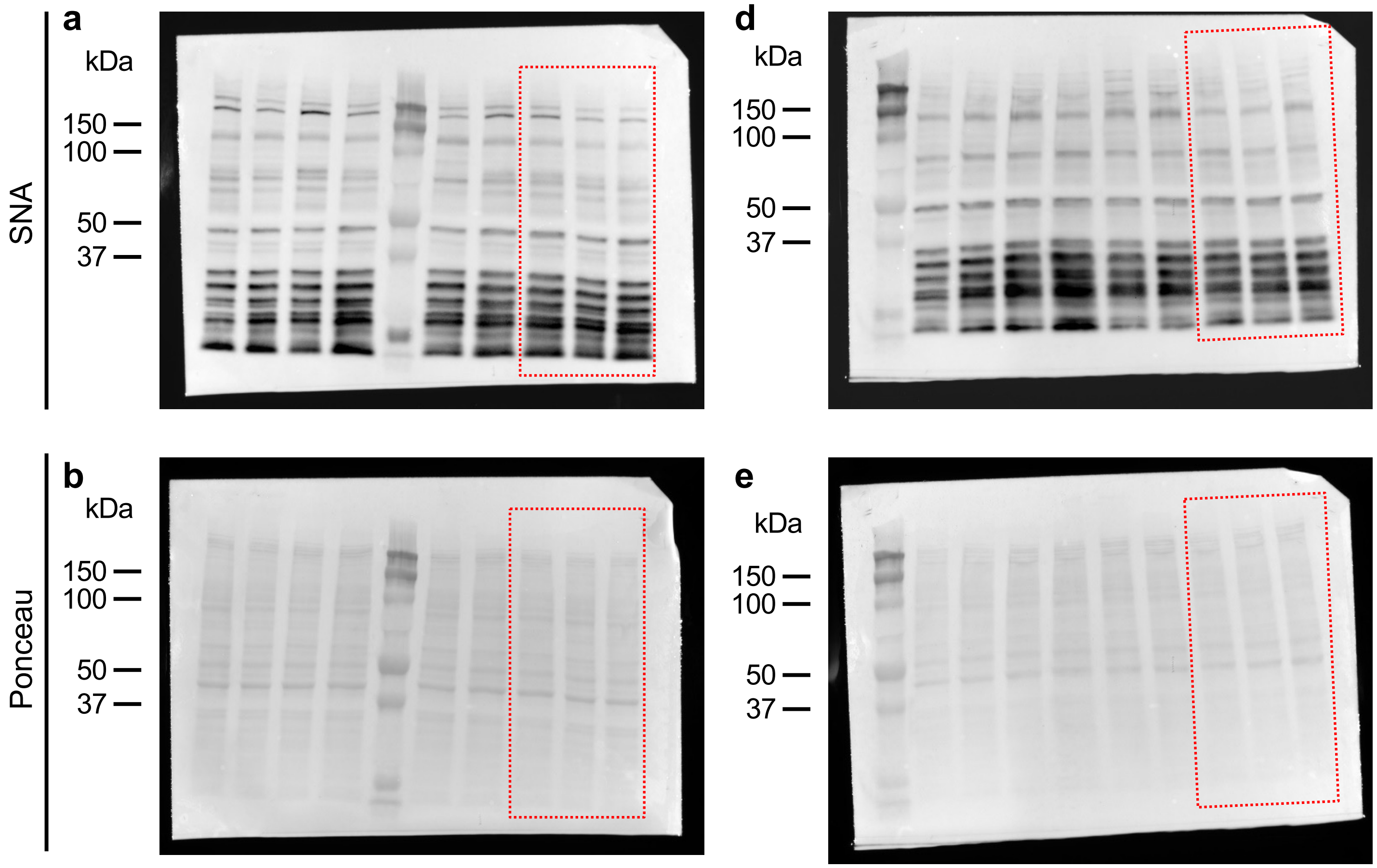

c Stage 2

Lane	Sample
1	Control$^{2-2}$
2	GNE$^{1-2}$
3	GNE$^{1-3}$
4	GNE$^{2-3}$
5	Ladder
6	GNE$^{2-2}$
7	Control$^{1-1}$
8	Control$^{1-2}$
9	GNE$^{2-1}$
10	GNE$^{1-1}$

Lanes: Left #1 → Right #10

f Stage 3

Lane	Sample
1	Ladder
2	Control$^{2-2}$
3	GNE$^{1-2}$
4	GNE$^{1-3}$
5	GNE$^{2-3}$
6	GNE$^{2-2}$
7	Control$^{1-1}$
8	Control$^{1-2}$
9	GNE$^{2-1}$
10	GNE$^{1-1}$

Lanes: Left #1 → Right #10

Supplemental Figure 12. ***Original sialic acid blots from control and GNEM patient-derived iMPCs portrayed in supplementary figure 7.*** (A and D) Raw blots used for the quantification of SNA and (B and E) total protein via ponceau staining. (C and F) Designation of samples loaded into each lane (for the two blots above each designation (e.g., c is for a and b)) starting with lane 1 on the far left of each blot and proceeding right. (A and B) Blots used for quantification of stage 2 SNA/Ponceau ratio from control and GNEM iMPC samples as represented in supplementary figure 7C. Red box = location of the cropped representative image found in supplementary figure 7A. (D and E) Blots used for quantification of stage 3 SNA/Ponceau ratio from control and GNEM iMPCs as represented in supplementary figure 7D. Red box = location of the cropped representative image found in supplementary figure 7B.

Line	Number of Clones	Mutations	Sex	Age (years)
Control[3]	1	N/A	Male	28
ACTA1	1	ACTA1 c.757G>C (p.Gly253Arg)	Male	71

Supplementary Table 1. **Features of additional male control and Nemaline myopathy patient (ACTA1) of which iPSC lines were derived.**

Line	Time Point	Filtered Cell Number
Control[1]	0	826
	1	1,764
	2	1,683
Control[2]	0	615
	1	1,861
	2	1,038
ACTA1	0	4,381
	1	606
	2	289
GNE[1]	0	9,064
	1	388
	2	201

Supplementary Table 2. **The total number of cells used at each time point in single-cell analyses.**

Ingenuity Canonical Pathways	-log(p-value)	Ratio	z-score
EIF2 Signaling	50.7	0.482	-5.25
Regulation of eIF4 and p70S6K Signaling	22.1	0.363	0.243
Actin Cytoskeleton Signaling	20.3	0.306	2.646
Integrin Signaling	20.2	0.324	3.302
ILK Signaling	20.1	0.333	1.05
Protein Ubiquitination Pathway	20	0.291	#NUM!
RHOGDI Signaling	20	0.321	-2.335
mTOR Signaling	19	0.316	0.6
Dilated Cardiomyopathy Signaling Pathway	17.1	0.351	1.342
Regulation of Actin-based Motility by Rho	16.8	0.388	2.722
Epithelial Adherens Junction Signaling	15.9	0.331	2.496
Signaling by Rho Family GTPases	14.9	0.261	2.101
Calcium Signaling	14.2	0.278	-2.117
Oxidative Phosphorylation	13.8	0.36	-3.162
Virus Entry via Endocytic Pathways	13.3	0.365	#NUM!
Remodeling of Epithelial Adherens Junctions	13.2	0.441	3.606
Germ Cell-Sertoli Cell Junction Signaling	12.9	0.292	#NUM!
RHOA Signaling	12.6	0.331	1.441
Hepatic Fibrosis / Hepatic Stellate Cell Activation	12.3	0.273	#NUM!
Caveolar-mediated Endocytosis Signaling	11.9	0.4	#NUM!
Mitochondrial Dysfunction	11.7	0.281	#NUM!
Coronavirus Pathogenesis Pathway	11.5	0.261	3.394
Estrogen Receptor Signaling	11.1	0.204	0.132
Semaphorin Neuronal Repulsive Signaling Pathway	10.8	0.285	-0.309
Actin Nucleation by ARP-WASP Complex	10.5	0.344	4.041

Supplementary Table 3. **Top 25 Canonical Pathways from Ingenuity Pathway Analysis from GNE.TP2 differentially expressed genes compared to Control1.TP2 and Control2.TP2.**

	Name	Company	Catalog	Dilution	Antibody Registry
Human Tissue	Rabbit Anti-p62/sequestosome Polyclonal Antibody	Abcam	ab91526	1:200	AB_2050336
Ectoderm	Sheep Anti-Human PAX6 Polyclonal Antibody	R&D Biosystems	AF8150	1:20	AB_2827378
	Mouse Anti-Human Nestin (Clone 10C2) Monoclonal Antibody	Stem Cell Technologies	60091	1:500	AB_2650581
Endoderm	Rabbit Anti-FoxA2 Monoclonal Antibody	Cell Signaling Technology	8186s	1:200	AB_10891055
	Goat Anti-Human SOX17 Polyclonal Antibody	R&D Biosystems	AF1924	1:20	AB_355060
Mesoderm	Rabbit Anti-Human CD31 Polyclonal Antibody	Abcam	ab32457	1:100	AB_726369
	Mouse Anti-Human CD56 (NCAM) (Clone HCD56) Monoclonal Antibody	Stem Cell Technologies	60021	1:200	AB_2891082
Pluripotency	Rabbit Anti-Oct4 Polyclonal Antibody	Abcam	ab19857	1:200	AB_445175
	Mouse Anti-SSEA4 (Clone MC813-70) Monoclonal Antibody	Abcam	ab16287	1:100	AB_778073
	Rabbit Anti-Human Nanog (Clone D73G4) XP Monoclonal Antibody	Cell Signaling Technology	4903	1:100	AB_10559205
	Mouse Anti-Human TRA-1-60(R) Monoclonal Antibody	R&D Biosystems	MAB4770	1:20	AB_2119062
Myogenic	Mouse Anti-Pax3 (Clone 274212) Monoclonal Antibody	R&D Biosystems	MAB2457	1:20	AB_2159398
	Rabbit Anti-Myf5 (Clone C-20) Polyclonal Antibody	Santa Cruz Biotechnology	SC-302	1:100	AB_631994
	Mouse Anti-MyoD (Clone 5.8A) Monoclonal Antibody	Santa Cruz Biotechnology	SC-32758	1:100	AB_627978
	Rabbit Anti-MyoG (Clone M-225) Polyclonal Antibody	Santa Cruz Biotechnology	SC-576	1:100	AB_2148908
	Mouse Anti-MF20 Monoclonal Antibody	Developmental Studies Hybridoma Bank	MF 20	1:2	AB_2147781
GNE Specific	Rabbit Anti-TDP-43 Polycloal Antibody	Proteintech	10782-2-AP	1:50	AB_615042
	Mouse Anti-LAMP-1 (Clone H4A3) Monoclonal Antibody	Santa Cruz Biotechnology	SC-20011	1:50	AB_626853
Secondaries and Nuclear stain	Donkey Anti-Rabbit Alexa Fluor 594	Invitrogen	A-21207	1:200	AB_141637
	Goat Anti-Mouse Alexa Fluor 594	Invitrogen	A-11005	1:300	AB_2534073
	Donkey Anti-Sheep Alexa Fluor 488	Abcam	ab150177	1:200	AB_2801320
	Donkey Anti-Goat Alexa Fluor 488	Invitrogen	A-11055	1:300	AB_2534102
	Donkey Anti-Mouse Alexa Fluor 488	Invitrogen	A-21202	1:300	AB_141607
	DAPI				
	NucBlue™ Fixed Cell ReadyProbes™ Reagent	Thermo	R37606	2 drops/1 mL PBS	

Supplementary Table 4. **Antibodies used in this study for immunofluorescence**.

	Name	Company	Catalog	Dilution	Antibody Registry
Loading Control Antibody	Mouse Anti-α-Tubulin (DM1A) Monoclonal Antibody	Cell Signaling Technology	3873	1:1,000	AB_1904178
Lectin Antibody	Sambucus Nigra Lectin (SNA, EBL), Biotinylated	Vector Laboratories	B-1305-2	1:1,000	AB_2336718
Autophagy Antibodies	Rabbit Anti-LC3B (D11) XP Monoclonal Antibody	Cell Signaling Technology	3868	1:1,000	AB_2137707
	Mouse Anti-LAMP-1 (Clone H4A3) Monoclonal Antibody	Santa Cruz Biotechnology	SC-20011	1:700	AB_626853
p38 Pathway Antibodies	Rabbit Anti-p38 MAPK Polyclonal Antibody	Cell Signaling Technology	9212	1:1,000	AB_330713
	Rabbit Anti-Phospho-p38 MAPK (Thr180/Tyr182) (D3F9) XP Monoclonal Antibody	Cell Signaling Technology	4511	1:1,000	AB_2139682
	Rabbit Anti-MAPKAPK-2 Antibody	Cell Signaling Technology	3042	1:1,000	AB_10694238
	Phospho-MAPKAPK-2 (Thr334) Antibody	Cell Signaling Technology	3041	1:1,000	AB_330726
Secondaries	Streptavidin, Peroxidase, ELISA Grade (Concentrate)	Vector Laboratories	SA-5014-1	3 μg/ml	AB_2336510
	Anti-rabbit IgG, HRP-linked Antibody	Cell Signaling Technology	7074	1:5,000	AB_2099233
	Anti-mouse IgG, HRP-linked Antibody	Cell Signaling Technology	7076	1:5,000	AB_330924

Supplementary Table 5. **Antibodies used in this study for western blotting**.
